# Causal association between gastroesophageal reflux disease and sepsis, and the mediating role of gut bacterial abundance, a Mendelian randomization study

**DOI:** 10.1097/MD.0000000000041631

**Published:** 2025-02-21

**Authors:** Zengyan Fu, Zhenhong Jiang, Fei Zhao, Tao Gou, Le Jiang

**Affiliations:** a Department of Emergency Medicine, The Affiliated Hospital of Hangzhou Normal University, Hangzhou, China.

**Keywords:** causal relationship, GERD, gut bacterial pathway abundance, mediation analysis, MR, sepsis

## Abstract

Gastroesophageal reflux disease (GERD), akin to sepsis, is mediated by inflammatory reactions and exhibits a strong correlation with intestinal dysbiosis. We sought to examine whether these associations reflect causality using the Mendelian randomization (MR) mediation analysis. Genetic data were obtained from genome-wide association studies. Two-sample MR were performed to evaluate the causal association, accompanied by sensitivity analyses. Reverse direction MR was undertaken to assess the potential for reverse causation. Then, mediation analysis was performed to evaluate the mediating effect of gut bacterial pathway abundance in this relationship. Genetic predisposition to GERD was significantly associated with sepsis [inverse variance weighting: odds ratio = 1.366, *P* = 2.13E-09, 95% confidence interval [CI] 1.233–1.513] and sepsis-related 28-day mortality (inverse variance weighting: odds ratio = 1.412, *P* = 6.64E-03, 95% CI 1.101–1.812). There is no convincing evidence for reverse causation. Gut bacterial pathway abundance (ARO.PWY..chorismate.biosynthesis.I) mediates the effect of GERD on sepsis (β = 0.036, 95% CI 0.004–0.067, *P* = .025), accounting for 11.406% of the total effect; Gut bacterial pathway abundance (PWY.7219..adenosine.ribonucleotides.de.novo.biosynthesis) mediates the effect of GERD on sepsis (β = 0.026, 95% CI −0.003 to 0.056, *P* = .083), accounting for 8.486% of the total effect; gut bacterial pathway abundance (ARO.PWY..chorismate.biosynthesis.I) mediates the effect of GERD on sepsis (28-day death) (β = 0.079, 95% CI 0.005–0.153, *P* = .036), accounting for 22.890% of the total effect; gut bacterial pathway abundance (TRNA.CHARGING.PWY..tRNA.charging) mediates the effect of GERD on sepsis (28-day death) (β = −0.066, 95% CI −0.140 to 0.007, *P* = .078), accounting for −19.171% of the total effect. The present MR study supported GERD as a causal risk factor of sepsis and sepsis-related 28-day mortality. Three specific gut bacterial pathway abundances were identified that played a partial mediating role in the aforementioned causal relationship between GERD and sepsis.

## 1. Introduction

Sepsis, an intricate and multifaceted medical syndrome, embodies a systemic inflammatory response that is orchestrated by an aberrant immune reaction to infection. This intricate interplay presents a dire and imminent threat to life, frequently precipitating life-threatening organ dysfunction. Presently, sepsis stands as one of the most prevalent and critically urgent acute illnesses.^[1]^ Recent research data underscores the overwhelming burden of sepsis, with an alarming annual global estimate of 47 to 50 million individuals succumbing to this devastating condition. Tragically, sepsis accounts for the lives lost of at least 11 million individuals annually, constituting a staggering 19.7% of all global deaths.^[2]^ In intensive care units, an alarming trend persists, with sepsis diagnoses annually afflicting millions of patients worldwide. Despite remarkable advancements in multi-organ support technologies and intensive care management strategies within these units, the mortality rate associated with sepsis remains stubbornly elevated, ranging from 30% to 50%.^[3]^

The gastrointestinal tract stands as a unique and multifaceted organ system within the human body, functioning not only as the cornerstone of the digestive process but also as a formidable component of the immune system.^[4]^ On the one hand, the gastrointestinal tract bears the dual burden of both endogenous and exogenous insults, resulting in compromised functionality and contributing significantly to the pathogenesis of sepsis. Conversely, it also serves as a pivotal intermediary, capable of exerting detrimental effects on distant organs via the release of inflammatory mediators and the translocation of bacteria and their toxic products.^[5]^

Gastroesophageal reflux disease (GERD) emerges as a prevalent clinical entity, intricately intertwined with upper gastrointestinal motility disorders. It encompasses a diverse array of symptoms and complications, stemming from the abnormal reflux of gastric contents into the esophagus. GERD manifests itself through a broad spectrum of clinical presentations, with heartburn and reflux serving as its cardinal symptoms. However, it is also capable of presenting with an array of atypical and extraesophageal manifestations, encompassing chest pain, abdominal distension, cough, dysphagia, and even asthma, underscoring its complex and multifaceted nature.^[6]^ The intricate pathogenesis of GERD continues to be a subject of intense research scrutiny, with contemporary knowledge pointing towards a complex interplay of diverse factors contributing to its onset. Notably, prior investigations have unveiled striking similarities between GERD and sepsis, both of which are fundamentally underpinned by inflammatory cascades and intricately intertwined with intestinal dysbiosis.^[7,8]^Extensive scientific investigations have unequivocally revealed a profound correlation between gut microbiota (GM) and its metabolic byproducts with respect to sepsis pathogenesis and progression.^[9–13]^ Several scholars have undertaken a comprehensive Mendelian randomization (MR) analysis to investigate the intricate relationship between intestinal flora and sepsis, yielding significant insights into their correlation.^[14]^ Nevertheless, factors encompassing the intricate composition of GM and their intricate interplay with various diseases may impede the progression of in-depth research endeavors. Consequently, the elusive causal relationships and the precise regulatory mechanisms that underpin the intricate interactions between GERD, intestinal microbiota, and sepsis warrant further scientific exploration.^[15]^

To address this gap in knowledge, we will employ a MR study to investigate the causal relationship between GERD and the occurrence and prognosis of sepsis, while concurrently elucidating the mediating role of GM in this intricate process. The MR approach leverages genetic variants, particularly single-nucleotide polymorphisms (SNPs), that are strongly associated with the exposure of interest, as instrumental variables (IVs) to assess the causal relationship between the exposure and the outcome. These genetic variants are randomly allocated at conception, present at birth, and remain relatively stable throughout the lifespan. Consequently, MR analysis resembles a randomized controlled trial in that it can minimize the influence of confounding factors and reverse causality, while also enabling the exploration of the mediating effects of intermediary factors between the exposure and the outcome.^[16]^ To the best of our knowledge, our study represents the first endeavor to elucidate the causal relationship between GERD and sepsis, while concurrently uncovering the mediatory role played by certain GM. Ultimately, our goal is to contribute strategic insights and causal evidence for the prevention and treatment of sepsis.

## 2. Materials and methods

### 2.1. Study design

**Figure 1.**
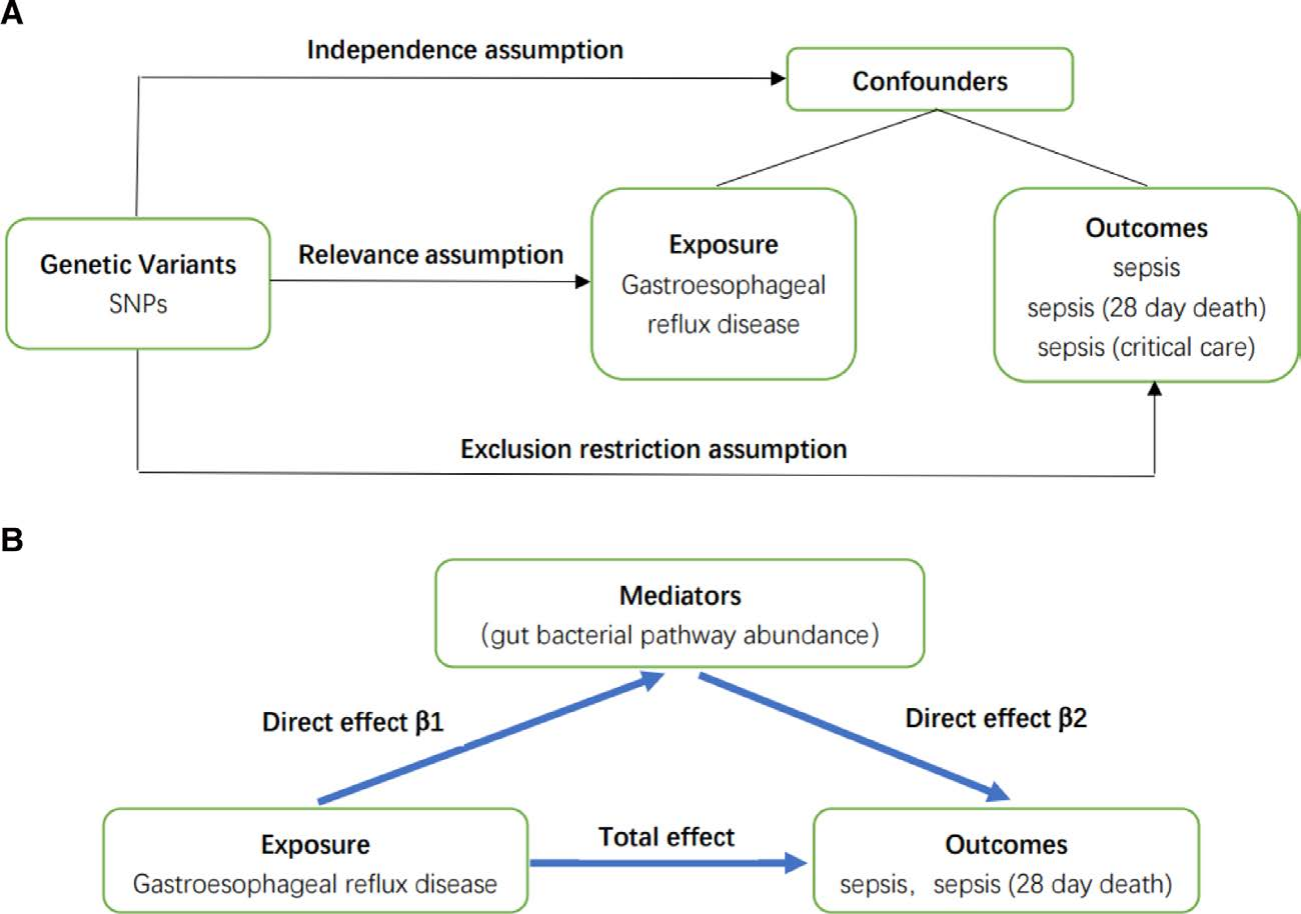
The diagram of our main MR study and mediator MR analysis. This diagram illustrates the potential causal pathway from GERD to sepsis, with gut bacterial pathway abundances as mediators. (A) Main MR analysis. (B) Mediator MR analysis. GERD = gastroesophageal reflux disease, MR = Mendelian randomization.

This study harnessed genetic variations as a tool to deduce causal linkages GERD and the outcome [Sepsis, Sepsis (28-day death), and Sepsis (critical care)]. The fundamental pillars of MR rest upon 3 core assumptions: (1) IV must exhibit a robust association with the exposure of interest. (2) These IVs must be unconfounded with respect to the exposure–outcome relationship, safeguarding against external influences that could bias the estimated causal effect. (3) Critically, the IVs should exclusively mediate their influence on the outcome solely through the exposure, and not via any alternative causal pathways. This exclusivity is paramount for the valid establishment of a causal link between the exposure and the outcome (Fig. 1A).

### 2.2. Data sources

All data on sepsis are sourced exclusively from genome-wide association study (GWAS) conducted by Hamilton F, focusing on European populations. Specifically, the data for Sepsis and Sepsis (28-day death) are derived from a rigorous analysis of 486,484 European individuals, with 11,643 cases of Sepsis and 1896 cases of Sepsis (28-day death) identified, respectively. Meanwhile, the data pertaining to Sepsis (critical care) are obtained from an equally meticulous analysis of 431,365 European individuals, encompassing 1380 cases.^[17]^ We obtained GWAS data on GERD from the research conducted by Ong JS, involving 602,604 European volunteers, among which 129,080 were cases [PMID: 34187846].^[18]^ Furthermore, we acquired information on Gut Bacterial Pathway Abundance from the GWAS analysis by Lopera-Maya EA, encompassing 7738 individuals of European descent [PMID: 35115690].^[19]^ (Table 1 and Table S1, Supplemental Digital Content, http://links.lww.com/MD/O423). All data in this study were obtained from GWAS, and the disease assessment and diagnosis for exposures, outcomes, and related variables all adhered to standard criteria. We extracted consolidated information from openly accessible databases (Open GWAS) which had received informed consent from the participants and had been granted ethical approval.

**Table 1:** Characteristics of data used in the Mendelian randomization study.

Trait	PMID	ncontrol	Sample size	ncase	Author
Sepsis	NA	474,841	486,484	11,643	Hamilton F
Sepsis (28 day death)	NA	484,588	486,484	1896	Hamilton F
Sepsis (critical care)	NA	429,985	431,365	1380	Hamilton F
GERD	34187846	473,524	602,604	129,080	Ong JS
Gut bacterial pathway abundance	35115690	NA	7738	NA	Lopera-Maya EA

GERD = gastroesophageal reflux disease.

### 2.3. IV selection criteria

In conducting our research, we painstakingly embarked on a multifaceted process for identifying the IVs employed, meticulously ensuring the robustness and veracity of our analytical framework. Initially, we rigorously extracted IVs from the dataset, applying a stringent threshold of (*P* < 5 × 10^-8^) to the data to guarantee that the selected variables exhibit statistically significant associations that are robustly correlated across the entire genomic landscape.^[20]^ To guarantee the independence among our IVs,^[21,22]^ we concurrently imposed a rigorous threshold for linkage disequilibrium at *r*^2^ < 0.001, alongside a clustering window of 10 Mb. Adhering to the prescribed operational procedures, we diligently identified and selected suitable IVs, subsequently subjecting the exposure data to a rigorous comparison with the outcome data. Moreover, to reinforce the robustness of our analysis, we methodically computed the F-statistic for each instrumental variable employing the formula F = β2/SE2, ensuring that all values surpassed the critical threshold of 10.^[23]^

When embarking on a MR analysis that explores mediating factors, we adopted a consistent threshold of (*P* < 5 × 10^-8^) for data selection. However, in instances where the MR analysis of mediating factors’ effects on outcomes faced a limitation in extracting sufficient SNPs using this threshold, we judiciously adjusted the threshold to (*P* < 5 × 10^-5^).^[20]^ This nuanced approach allows us to extract IVs that are significantly associated across the entire genome. As per the previously established criteria, we established a stringent threshold for linkage disequilibrium at *r*^2^ < 0.001, coupled with a clustering window of 10 Mb.^[21,22]^ Furthermore, we rigorously applied the formula F = β2/SE2, ensuring that the F-statistic for all IVs exceeded 10.^[23]^

### 2.4. MR analysis

We leveraged the “TwoSampleMR” R package, particularly the “mr()” function, to perform a rigorous two-sample MR analysis on our dataset. Among the myriad of methods at our disposal, we opted for the inverse variance-weighted (IVW) method as our primary analytical tool, given its superior statistical efficiency under the fulfillment of the 3 cornerstone assumptions of MR.^[20,24]^ To comprehensively evaluate and mitigate the potential biases arising from horizontal pleiotropy and heterogeneity, we additionally utilized the weighted median method and the MR-Egger method as complementary strategies. In scenarios where horizontal pleiotropy is present to a certain extent, the MR-Egger method proves to be a reliable analytical tool, while the weighted median method can offer robust estimates when the proportion of invalid IVs does not exceed 50%.^[25,26]^ If a large number of SNPs are screened out, we will not seek proxies for the missing SNPs. We employed the odds ratio (OR) value as a metric to rigorously evaluate the existence of a potential causal relationship within our data.

### 2.5. Sensitivity analyses

To mitigate potential biases in our significant findings, we conducted comprehensive sensitivity analyses. When the resulting *P*-value surpassed the threshold of .05, we interpreted this as compelling evidence that the influence of horizontal pleiotropy on our findings can be confidently discounted.^[27,28]^ To assess the presence of heterogeneity in our data, we utilized the Cochran *Q* test. Consistent with standard practice, when the Q-statistic (QP) exceeded 0.05, we concluded that the data were not significantly impacted by heterogeneity. Furthermore, to investigate potential biases stemming from specific SNPs, we conducted a leave-one-out analysis, particularly when the number of SNPs exceeded 2, to ensure the robustness of our findings.^[29]^ All the aforementioned analyses were carried out using the “TwoSampleMR” R package (version 0.5.7) and “MRPRESSO” package in R (version 4.3.1).

### 2.6. Mediator MR analysis

To elucidate the potential mediatory role of GM pathway abundance in the intricate relationship between GERD and sepsis, we undertook a rigorous two-step MR analysis. In this framework, β1 quantifies the influence of GERD on the abundance of GM pathways, while β2 captures the subsequent effect of these microbial pathways on the onset of sepsis. Additionally, β3 directly measures the effect of GERD on sepsis, bypassing the microbial intermediate. To quantify the extent to which GM pathway abundance mediates the GERD-sepsis relationship, we estimated the percentage of the mediating effect by calculating the ratio of the indirect effect (mediated through GM) to the total effect (β1 × β2/β3) (Fig. 1B).

## 3. Results

### 3.1. Causal association between GERD and sepsis

All the data samples of GERD, sepsis, and gastrointestinal bacterial abundance mentioned in the previous data sources were used for MR analysis. In our study, we initially assessed the influence of GERD on sepsis across varying degrees of severity. After data processing, we identified a total of 80 eligible SNPs that match GERD, sepsis, sepsis (28-day death), and sepsis (critical care) (Table S2, Supplemental Digital Content, http://links.lww.com/MD/O423). The F-statistic, which serves as a robust metric for quantifying the strength of the association between genetic variation and the exposure of interest, ranged from 207 to 670 (Table S3, Supplemental Digital Content, http://links.lww.com/MD/O423). This observation underscores the absence of concern regarding the risk of weak instrument variables in our analytical framework.

**Figure 2.**
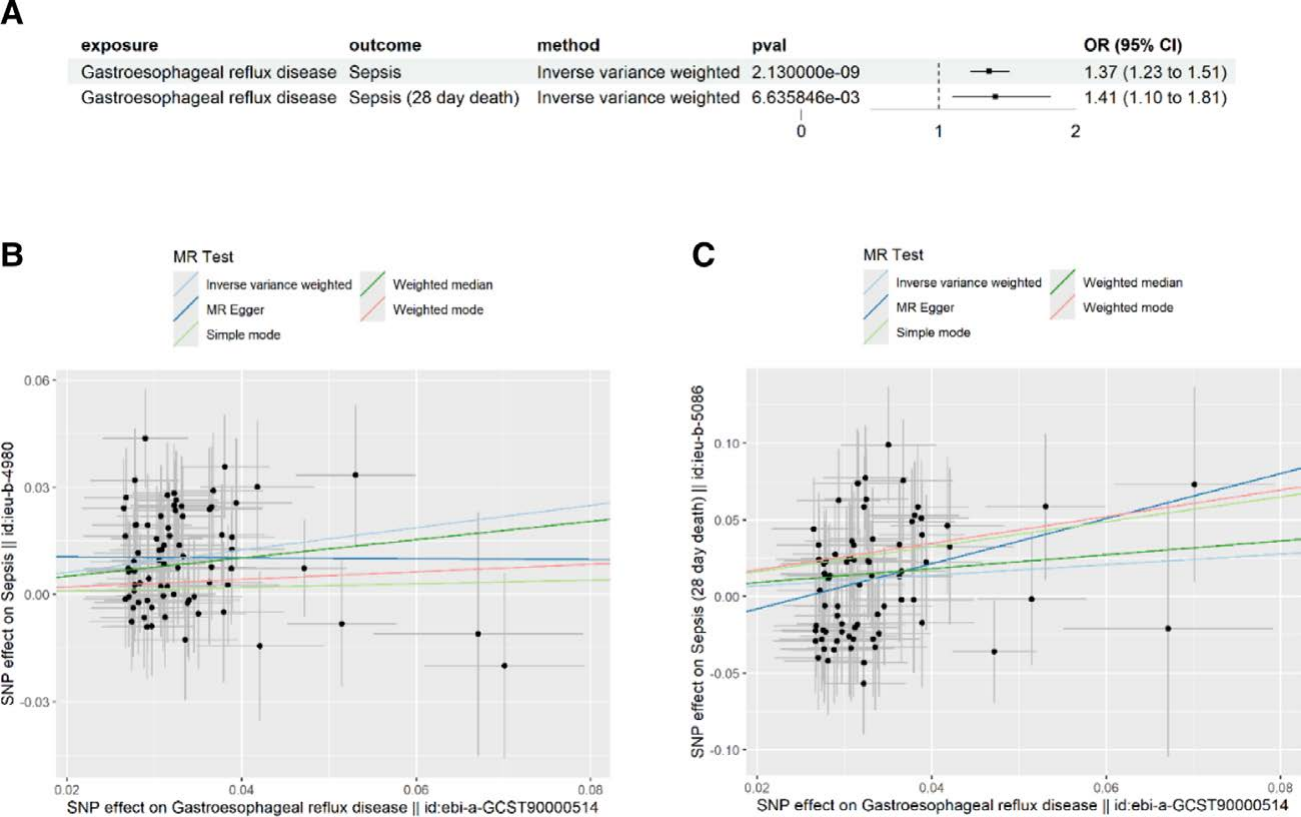
(A) The influence of GERD on sepsis and sepsis (28-day death). (B) Scatter plot showing the outcomes of GERD on sepsis. (C) Scatter plot showing the outcomes of GERD on sepsis with respect to 28-day mortality. GERD = gastroesophageal reflux disease, MR = Mendelian randomization, OR = odds ratio, SNP = single-nucleotide polymorphism.

The results of the impact of GERD on sepsis are presented in Figure 2.

In our results, we observed a significant causal relationship between GERD and the occurrence of sepsis (IVW: OR = 1.366, *P* = 2.13E-09, 95% CI: 1.233–1.513; MR-Egger: *P* = .964). This finding remained robust in sensitivity analyses (Table S4, Supplemental Digital Content, http://links.lww.com/MD/O423), unaffected by heterogeneity and horizontal pleiotropy. Additionally, reverse causality analysis indicated the absence of reverse causality (Table S5, Supplemental Digital Content, http://links.lww.com/MD/O423), and leave-one-out analysis (Figure S1, Supplemental Digital Content, http://links.lww.com/MD/O425) did not reveal any outliers. We then observed a substantial causal relationship between GERD and the occurrence of sepsis-related 28-day mortality (IVW: OR = 1.412, *P* = 6.64E-03, 95% CI: 1.101–1.812). This finding remained robust in sensitivity analyses (Table S4, Supplemental Digital Content, http://links.lww.com/MD/O423), unaffected by heterogeneity and horizontal pleiotropy. Furthermore, reverse causality analysis indicated the absence of reverse causality (Table S5, Supplemental Digital Content, http://links.lww.com/MD/O423), and leave-one-out analysis (Figure S2, Supplemental Digital Content, http://links.lww.com/MD/O425) did not reveal any outliers. It is noteworthy that our findings indicate no significant influence of GERD on sepsis outcomes in the intensive care setting (Table S4, Supplemental Digital Content, http://links.lww.com/MD/O423).

### 3.2. The mediating effect of gut bacterial pathway abundance

#### 3.2.1. The MR analysis of the impact of GERD on gut bacterial pathway abundance

**Figure 3.**
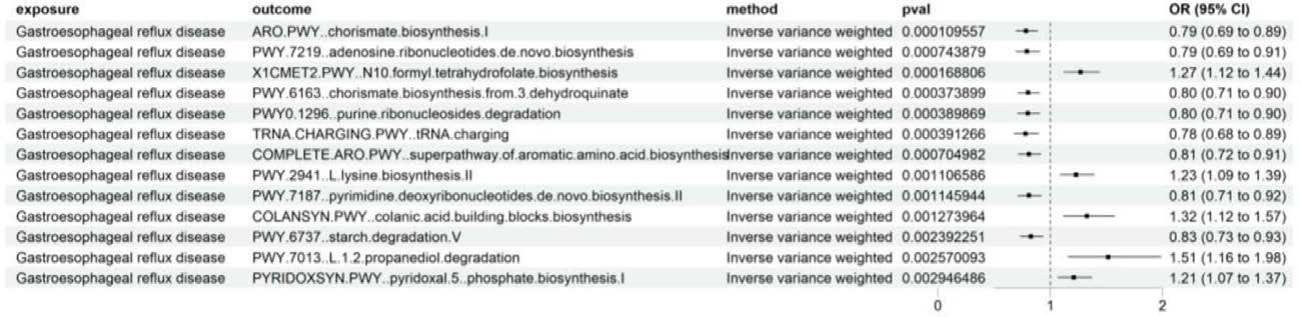
The impact of GERD on gut bacterial pathway abundance. GERD = gastroesophageal reflux disease, OR = odds ratio.

The results of the impact of GERD on gut bacterial pathway abundance are shown in Figure 3.

We have conducted a comprehensive assessment of the influence of GERD on the gut bacterial pathway abundance. We employed the F-statistic as a robust indicator to quantify the strength of the association between genetic variation and exposure. Our analysis revealed that the F-statistic values span from 207 to 670 (Table S3, Supplemental Digital Content, http://links.lww.com/MD/O423), unequivocally demonstrating the absence of any risk associated with weak instrument variables.

Upon analysis, we have identified thirteen positive findings. They are respectively gut bacterial pathway abundance ARO.PWY..chorismate.biosynthesis.I (IVW: OR = 0.785, *P* = 1.10E-04, FDR-pval = 0.022,95% CI: 0.694–0.887); X1CMET2.PWY..N10.formyl.tetrahydrofolate.biosynthesis (IVW: OR = 1.269, *P* = 1.69E-04, FDR-pval = 0.017, 95% CI: 1.121–1.437); PWY.6163..chorismate.biosynthesis.from.3.dehydroquinate (IVW: OR = 0.800, *P* = 3.74E-04, FDR-pval = 0.026, 95% CI: 0.708–0.905); PWY0.1296..purine.ribonucleosides.degradation (IVW: OR = 0.800, *P* = 3.90E-04, FDR-pval = 0.020, 95% CI: 0.707–0.905); TRNA.CHARGING.PWY..tRNA.charging (IVW: OR = 0.778, *P* = 3.91E-04, FDR-pval = 0.016, 95% CI: 0.677–0.894); COMPLETE.ARO.PWY..superpathway.of.aromatic.amino.acid.biosynthesis (IVW: OR = 0.809, *P* = 7.05E-04, FDR-pval = 0.024, 95% CI: 0.715–0.914); PWY.7219..adenosine.ribonucleotides.de.novo.biosynthesis (IVW: OR = 0.792, *P* = 7.44E-04, FDR-pval = 0.022, 95% CI: 0.692–0.907); PWY.2941..L.lysine.biosynthesis.II (IVW: OR = 1.228, *P* = 1.11E-03, FDR-pval = 0.028, 95% CI: 1.085–1.389); PWY.7187..pyrimidine.deoxyribonucleotides.de.novo.biosynthesis.II (IVW: OR = 0.808, *P* = 1.15E-03, FDR-pval = 0.026, 95% CI: 0.710–0.919); COLANSYN.PWY..colanic.acid.building.blocks.biosynthesis (IVW: OR = 1.324, *P* = 1.27E-03, FDR-pval = 0.026, 95% CI: 1.116–1.570)PWY.6737..starch.degradation.V (IVW: OR = 0.827, *P* = 2.39E-03, FDR-pval = 0.045, 95% CI: 0.731–0.935); PWY.7013..L.1.2.propanediol.degradation (IVW: OR = 1.515, *P* = 2.57E-03, FDR-pval = 0.044, 95% CI: 1.156–1.984); PYRIDOXSYN.PWY..pyridoxal.5..phosphate.biosynthesis.I (IVW: OR = 1.208, *P* = 2.95E-03, FDR-pval = 0.046, 95% CI: 1.066–1.368). The sensitivity analysis (Table S4, Supplemental Digital Content, http://links.lww.com/MD/O423) revealed that the aforementioned results were generally able to exclude the influence of heterogeneity and pleiotropy, with the exception of gut bacterial pathway abundance (COLANSYN.PWY.colanic.acid.building.blocks.biosynthesis), for which the influence of heterogeneity could not be fully excluded, but the influence of pleiotropy was excluded. The leave-one-out analysis of the above results did not reveal any abnormal values, as detailed in the Figures S3–S15, Supplemental Digital Content, http://links.lww.com/MD/O425. The scatter plot of the above results can be found in the Files S1–S13, Supplemental Digital Content, http://links.lww.com/MD/O424.

#### 3.2.2. The MR analysis of the impact of gut bacterial pathway abundance on sepsis

**Figure 4.**
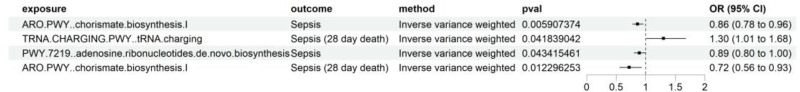
The influence of gut bacterial pathway abundance on sepsis and sepsis (28-day death). OR = odds ratio.

The results of the impact of gut bacterial pathway abundance on sepsis are shown in Figure 4.

Building upon the aforementioned results, we have further undertaken an MR analysis focusing on the positive associations to evaluate the influence of gut bacterial pathway abundance on sepsis. We employed the F-statistic as a robust indicator to quantify the strength of the association between genetic variation and exposure. Our analysis revealed that the F-statistic values span from 19 to 161 (Table S3, Supplemental Digital Content, http://links.lww.com/MD/O423), unequivocally demonstrating the absence of any risk associated with weak instrument variables.

The results we obtained are as follows: the influence of gut bacterial pathway abundance (ARO.PWY..chorismate.biosynthesis.I) on sepsis (IVW: OR = 0.863, *P* = 5.91E-03, 95% CI: 0.778–0.959), the influence of gut bacterial pathway abundance (ARO.PWY..chorismate.biosynthesis.I) on sepsis (28-day death) (IVW: OR = 0.721, *P* = .012, 95% CI: 0.559–0.932), the influence of gut bacterial pathway abundance (PWY.7219..adenosine.ribonucleotides.de.novo.biosynthesis) on sepsis (IVM: OR = 0.893, *P* = .043, 95% CI: 0.799–0.997), the influence of gut bacterial pathway abundance (TRNA.CHARGING.PWY..tRNA.charging) on sepsis (28-day death) (IVW: OR = 1.302, *P* = .042, 95% CI: 1.010–1.678). The sensitivity analysis (Table S4, Supplemental Digital Content, http://links.lww.com/MD/O423) revealed that the aforementioned results were able to exclude the influence of heterogeneity and pleiotropy. The leave-one-out analysis of the above results did not reveal any abnormal values, as detailed in the Figures S16–S19, Supplemental Digital Content, http://links.lww.com/MD/O425. The scatter plot of the above results can be found in the Files S14–S17, Supplemental Digital Content, http://links.lww.com/MD/O424.

#### 3.2.3. Mediator MR analysis

Having established the mediating effects of gut bacterial pathway abundance in the relationship between GERD and sepsis, we proceeded to conduct a mediation analysis on the gut bacterial pathway abundance, with the aim of quantifying its mediating proportion in the influence of GERD on sepsis. The results we obtained are as follows (Table S6, Supplemental Digital Content, http://links.lww.com/MD/O423): gut bacterial pathway abundance (ARO.PWY..chorismate.biosynthesis.I) mediates the effect of GERD on sepsis (β = 0.036, 95% CI: 0.004–0.067, *P* = .025), accounting for 11.406% of the total effect; gut bacterial pathway abundance (PWY.7219..adenosine.ribonucleotides.de.novo.biosynthesis) mediates the effect of GERD on sepsis (β = 0.026, 95% CI: −0.003 to 0.056, *P* = .083), accounting for 8.486% of the total effect; gut bacterial pathway abundance (ARO.PWY..chorismate.biosynthesis.I) mediates the effect of GERD on sepsis (28-day death) (β = 0.079, 95% CI: 0.005–0.153, *P* = .036), accounting for 22.890% of the total effect; gut bacterial pathway abundance (TRNA.CHARGING.PWY..tRNA.charging) mediates the effect of GERD on sepsis (28-day death) (β = −0.066, 95% CI: −0.140 to 0.007, *P* = .078), accounting for −19.171% of the total effect.

## 4. Discussion

To date, sepsis remains one of the most threatening diseases to human health worldwide. Despite advancements in clinical technology and basic research, which have led to a decline in sepsis mortality over the past decade, sepsis continues to pose as one of the most challenging emergency critical illnesses in the field of critical care medicine due to its intricate pathogenesis and complex pathophysiological processes.^[30]^ The precise pathogenesis of this condition remains largely elusive to date, and its progression encompasses intricate interactions among multiple organs and systems, with the gastrointestinal tract being a particularly distinctive component in this complex interplay.^[31]^In the history of medicine, the intestine has also been considered as the primary linking organ for sepsis and multiple organ dysfunction syndrome.^[32,33]^ This study delves into the relationship between GERD, GM, and sepsis using the MR approach. The MR analysis employed in this research is a methodology that utilizes genetic variations as IVs to assess causality. Based on Mendel laws, which postulate the random distribution of genes, this approach mitigates the issues of confounding factors and reverse causality commonly encountered in traditional epidemiological studies.^[34]^ We successfully identified a causal relationship between GERD and the occurrence of sepsis (IVW: OR = 1.366, *P* = 2.13E-09, 95% CI: 1.233–1.513), as well as an independent and causal association with an increased risk of 28-day mortality in sepsis patients (IVW: OR = 1.412, *P* = 6.64E-03, 95% CI: 1.101–1.812). Reverse analysis revealed that these quantitative results had no impact on GERD. Additionally, the results indicated that GERD did not influence the need for intensive care in sepsis patients.

According to statistics, the global prevalence of GERD is 13.98%, with an annual upward trend.^[35,36]^ While the exact mechanisms by which GERD may lead to sepsis are not fully understood, several studies are underway to elucidate this connection. Impairment of mucosal barrier function is a crucial factor contributing to the pathogenesis of GERD.^[37]^ The mucosal barrier functions as a vital separator, dividing intestinal immune cells from the microbial community and subsequently reducing intestinal permeability. Compromised intestinal permeability and mucosal immune function can lead to the translocation of pathogenic microorganisms, which triggers an excessive production of inflammatory factors. This inflammatory cascade ultimately culminates in the onset or exacerbation of sepsis, underscoring the significance of maintaining a healthy mucosal barrier.^[38]^ Based on our research findings, we hypothesize that GERD may contribute to bacterial translocation by enhancing intestinal permeability and impairing intestinal mucosal immune function, thereby triggering and exacerbating sepsis. Studies have revealed that patients with GERD exhibit distinct bacterial abundance compared to healthy individuals, accompanied by significantly elevated levels of certain proinflammatory cytokines, including interleukin-4, interleukin-1β, and tumor necrosis factor-α.^[39,40]^ Additionally, in patients with GERD, an increased abundance of gram-negative bacilli has been observed, which leads to an upregulation of inflammatory cytokines such as interleukin-18, tumor necrosis factor-α, and cyclooxygenase-2. Notably, probiotics exhibit a significant inhibitory effect on the inflammatory responses induced by these factors.^[41]^ Concurrently, GERD triggers the release of gastric and duodenal contents, including gastric acid, pepsin, trypsin, and bile, which stimulate inflammatory immune cells, leading to the production of various inflammatory mediators.^[42,43]^ This suggests that significant alterations occur in the composition and distribution of intestinal microbiota in patients with GERD, accompanied by elevated levels of proinflammatory cytokines. Consequently, this leads to an increase in the systemic inflammatory response status, which may underlie the pathogenesis and progression of sepsis. This finding aligns with the results of our study.

The GM plays a pivotal role in influencing the onset of numerous diseases and has emerged as a crucial focus for understanding the pathogenesis of gastrointestinal disorders. In recent years, numerous studies have demonstrated the significance of GM diversity in GERD and its role in the progression of sepsis.^[44–46]^ Imbalance in GM can lead to an increase in gram-negative bacteria, accompanied by a rise in lipopolysaccharide, which serves as the primary component of the cell wall of gram-negative bacteria. Lipopolysaccharide, being a prototypical endotoxin, promotes the secretion of inflammatory cytokines,^[47–49]^ and excessive oxidative stress and inflammatory response are considered significant contributors to the development of multiple organ failure caused by sepsis. The intestinal microbiota represents the largest microbial ecosystem in humans, comprising over 500 species with a total population reaching up to 10^14^ microorganisms,^[50]^ there exists considerable variation in the intestinal microbiota in terms of quantity, species diversity, and functional capabilities. Dysbiosis of the GM, characterized by an increase in pathogenic bacteria, may contribute to the development of bacterial sepsis.^[51]^ In the context of a protective commensal microbiota, pathogenic bacteria within the gut of a healthy host may remain dormant and nonpathogenic. However, the absence of this protective microbial community can facilitate the excessive growth of pathogenic bacteria.^[52,53]^ Previous studies have indicated that the GM of septic patients is characterized by reduced diversity, with decreased relative abundance of taxa such as Bacteroidetes and Firmicutes, as well as a decrease in the number of commensal bacteria like Faecalibacterium, Lactobacillus, and Ruminococcus. Additionally, there is an overgrowth of potential pathogens, including Enterobacteriaceae, Enterococcus, and Staphylococcus.^[54–57]^ Similarly, significant alterations in the microbiota may be associated with the progression of sepsis.^[58]^ Studies have demonstrated that GM dysfunction serves as a major risk factor for the development of late-onset sepsis.^[59,60]^ Du et al^[10]^ discovered that an imbalance in GM is associated with increased mortality, suggesting that GM can serve as a prognostic indicator for sepsis. In our assessment of the mediating effects of GM abundance between GERD and sepsis, we found that GERD is correlated with the abundance of thirteen gut bacterial pathways. Among these, 3 gut bacterial pathways potentially mediate the occurrence of sepsis and 28-day mortality in sepsis patients. These 3 pathways are: ARO.PWY..chorismate.biosynthesis.I, PWY.7219..adenosine.ribonucleotides.de.novo.biosynthesis, and TRNA.CHARGING.PWY..tRNA.charging.

Certainly, our study has several limitations that warrant acknowledgments. Firstly, the data used in this study were exclusively from European populations, which may introduce racial biases in the observed causal relationships. Secondly, our analysis focused on the genus level of bacterial taxa, rather than more specific levels such as strains or species, limiting the ability to obtain more detailed and precise results. Lastly, larger-scale GWAS data are needed to identify additional genetic variants for use in MR studies, which could further strengthen our findings.

## 5. Conclusion

Utilizing MR analysis, our study has validated the hypothesis of a causal association between GERD and sepsis, including among sepsis patients who succumb within 28 days. Additionally, we have uncovered specific GM that contribute to the development of sepsis in individuals with GERD, thereby reinforcing the conjecture that GM plays a mediating role in sepsis pathogenesis. Our findings provide valuable insights into the role of intestinal microbiota in sepsis management, including reducing the risk of sepsis, minimizing mortality rates, and improving sepsis prognosis.

## Acknowledgments

The authors acknowledge the participants and investigators of the original studies for sharing the GWAS data that were used in this study.

## Author contributions

**Conceptualization:** Zengyan Fu, Le Jiang.

**Data curation:** Zengyan Fu.

**Formal analysis:** Zengyan Fu.

**Investigation:** Fei Zhao.

**Methodology:** Fei Zhao.

**Project administration:** Le Jiang.

**Resources:** Zhenhong Jiang.

**Supervision:** Le Jiang.

**Software:** Zhenhong Jiang.

**Validation:** Le Jiang.

**Visualization:** Tao Gou.

**Writing – original draft:** Zengyan Fu.

**Writing – review & editing:** Zengyan Fu, Le Jiang.

## Supplementary Material


