# Supplementary material for: Causal association between gastroesophageal reflux disease and sepsis, and the mediating role of gut bacterial abundance, a Mendelian randomization study
Source: Medicine (Baltimore). 2025 Feb 21;104(8):e41631. doi: 10.1097/MD.0000000000041631 (PMC11857025; doi:10.1097/MD.0000000000041631)
Supplement: Supplementary file 2 [file medi-104-e41631-s002.pdf]

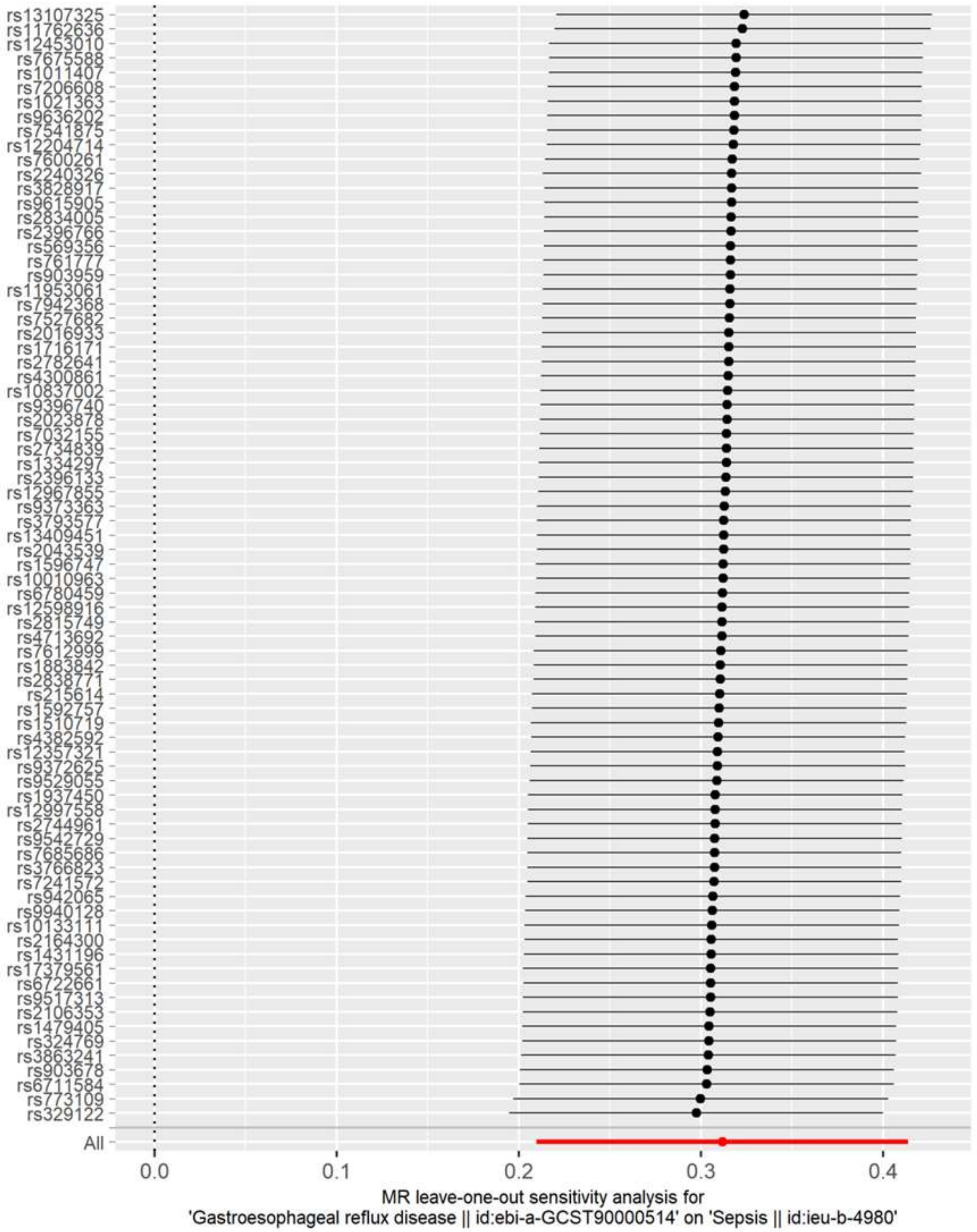

Supplementary Figure 1: Leave-one-out plot of SNPs related to GERD and sepsis.

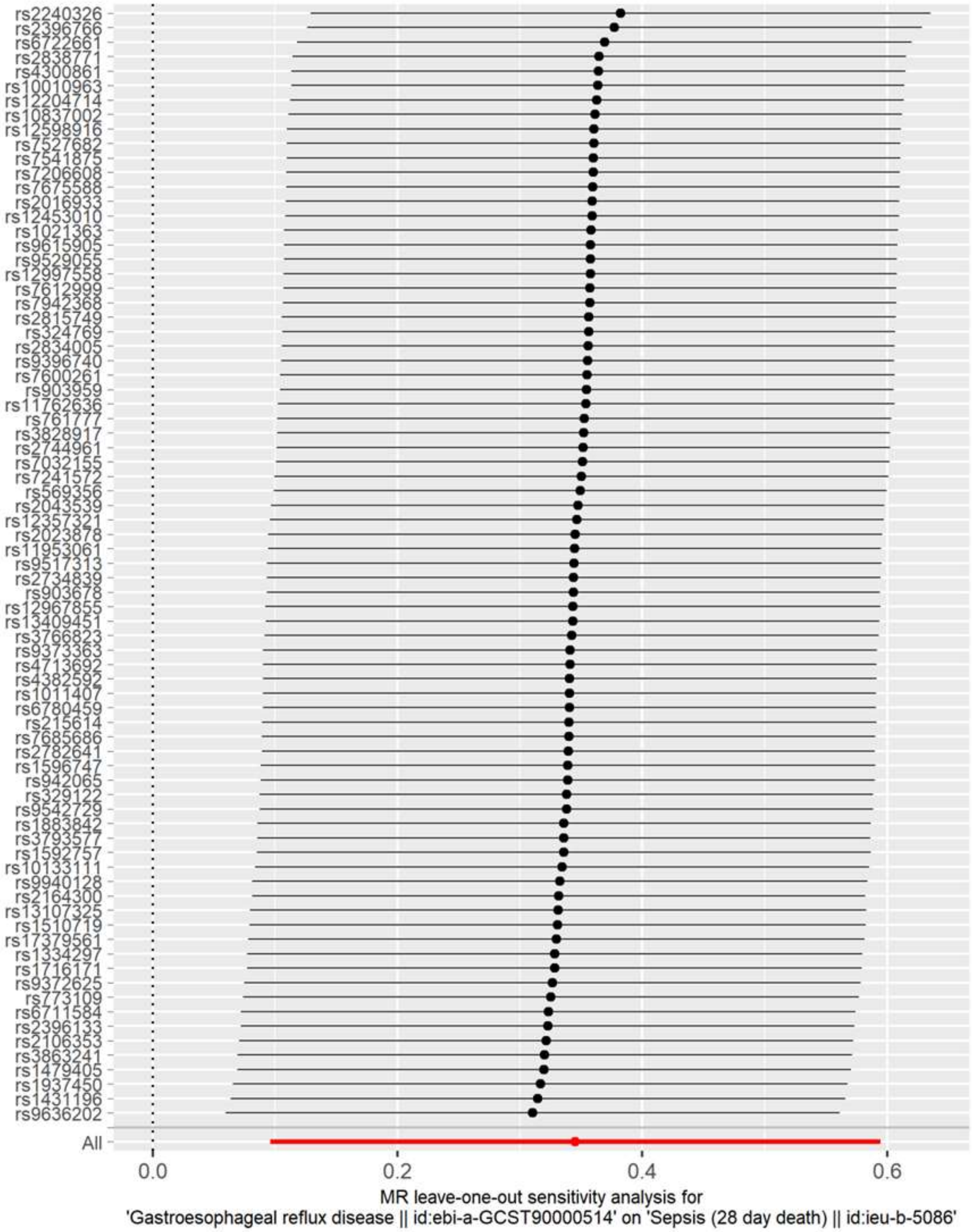

Supplementary Figure 2: Leave-one-out plot of SNPs related to GERD and sepsis (28 day death).

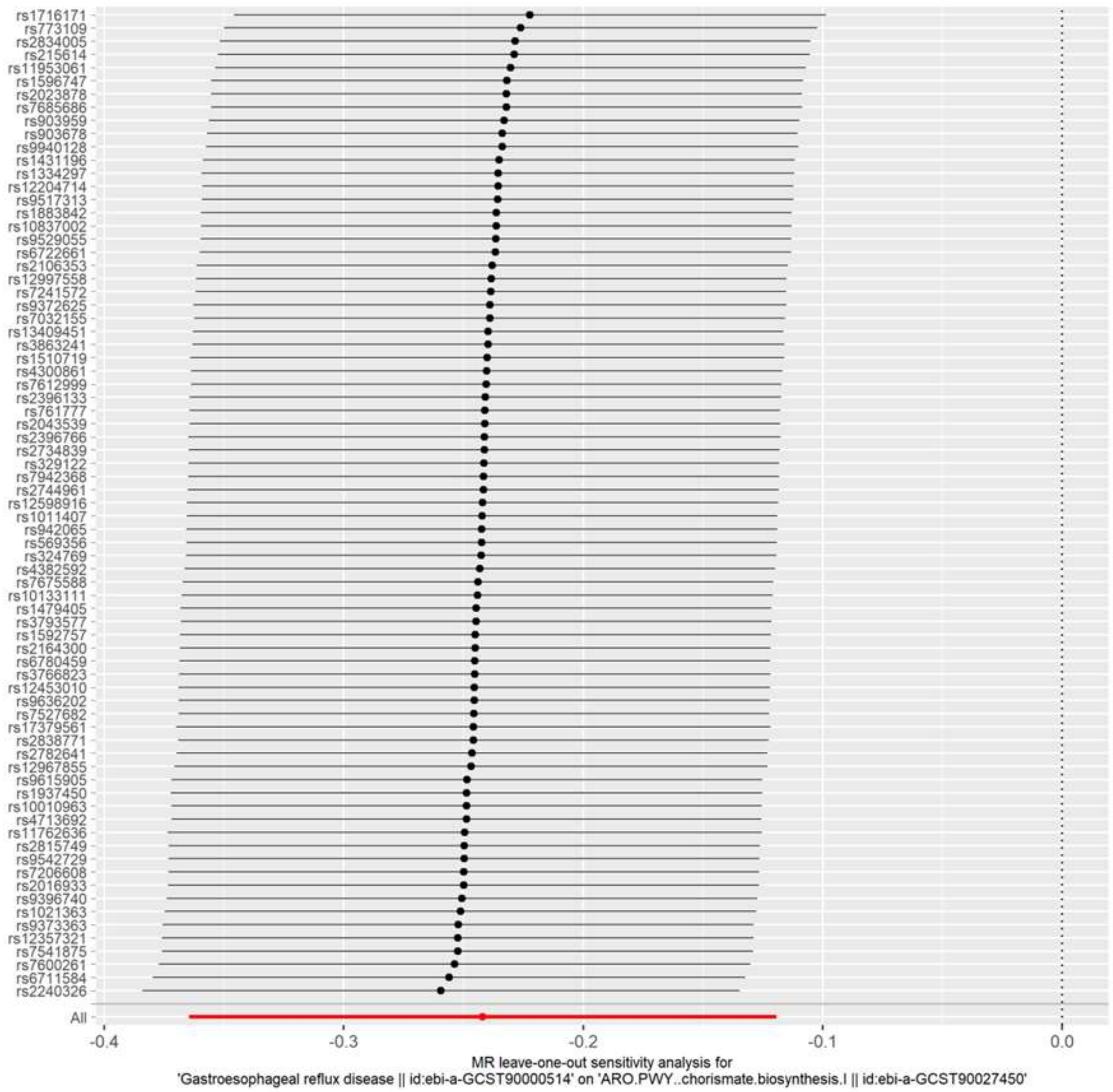

Supplementary Figure 3: Leave-one-out plot of SNPs related to GERD and gut bacterial pathway abundance (ARO.PWY..chorismate.biosynthesis.I).

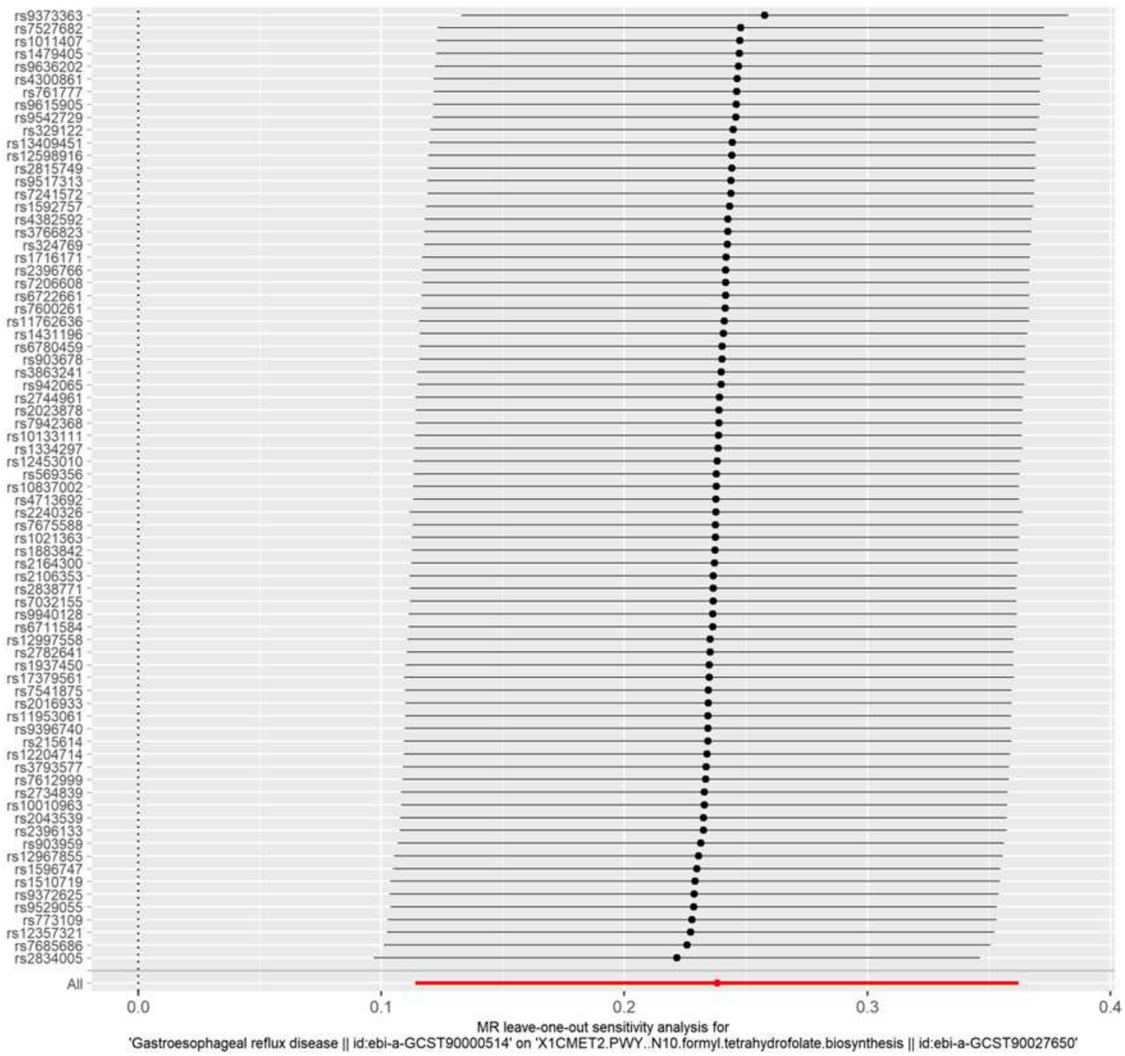

Supplementary Figure 4: Leave-one-out plot of SNPs related to GERD and gut bacterial pathway abundance (X1CMET2.PWY..N10.formyl.tetrahydrofolate.biosynthesis).

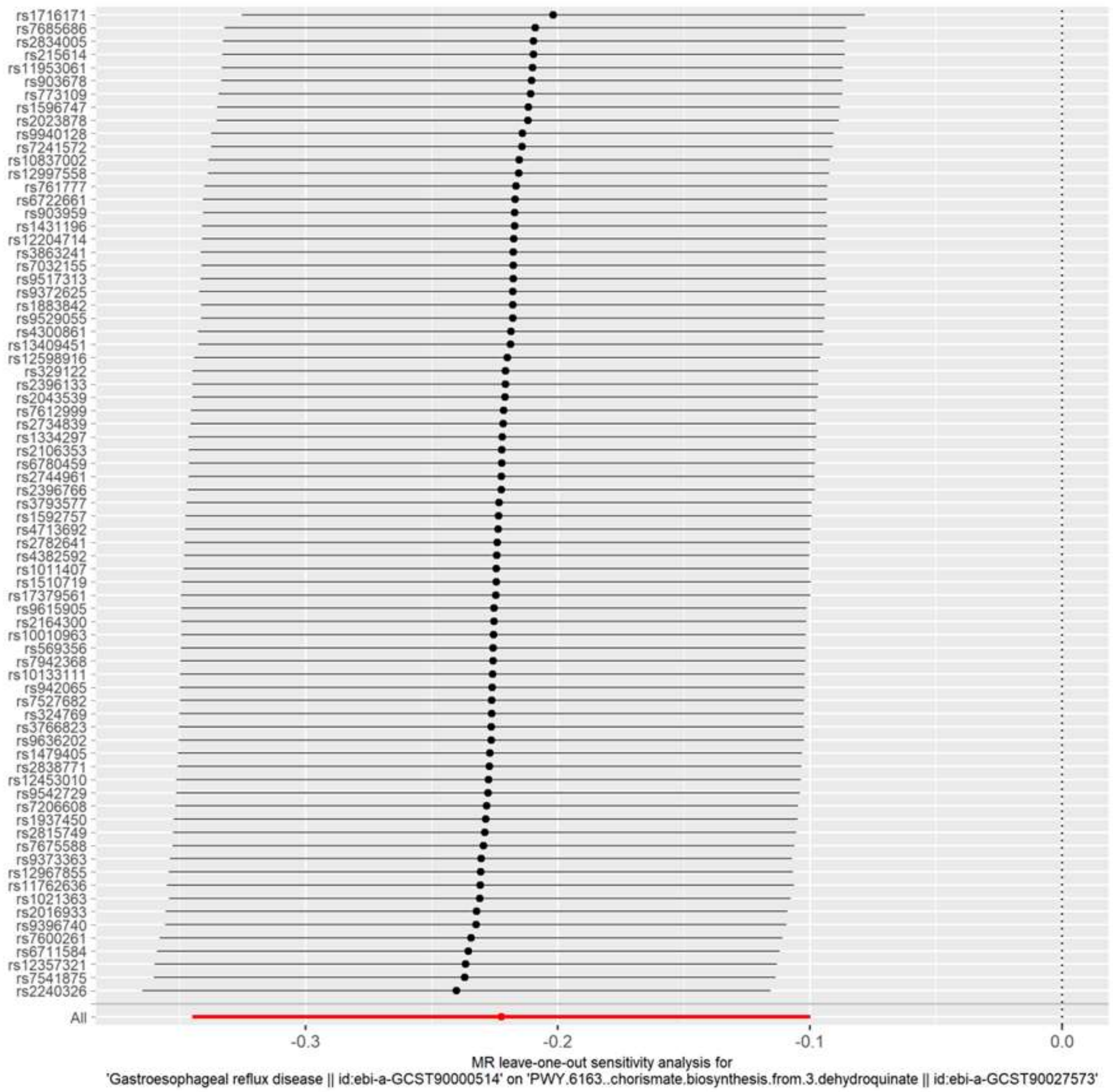

Supplementary Figure 5: Leave-one-out plot of SNPs related to GERD and gut bacterial pathway abundance (PWY.6163..chorismate.biosynthesis.from.3.dehydroquinate).

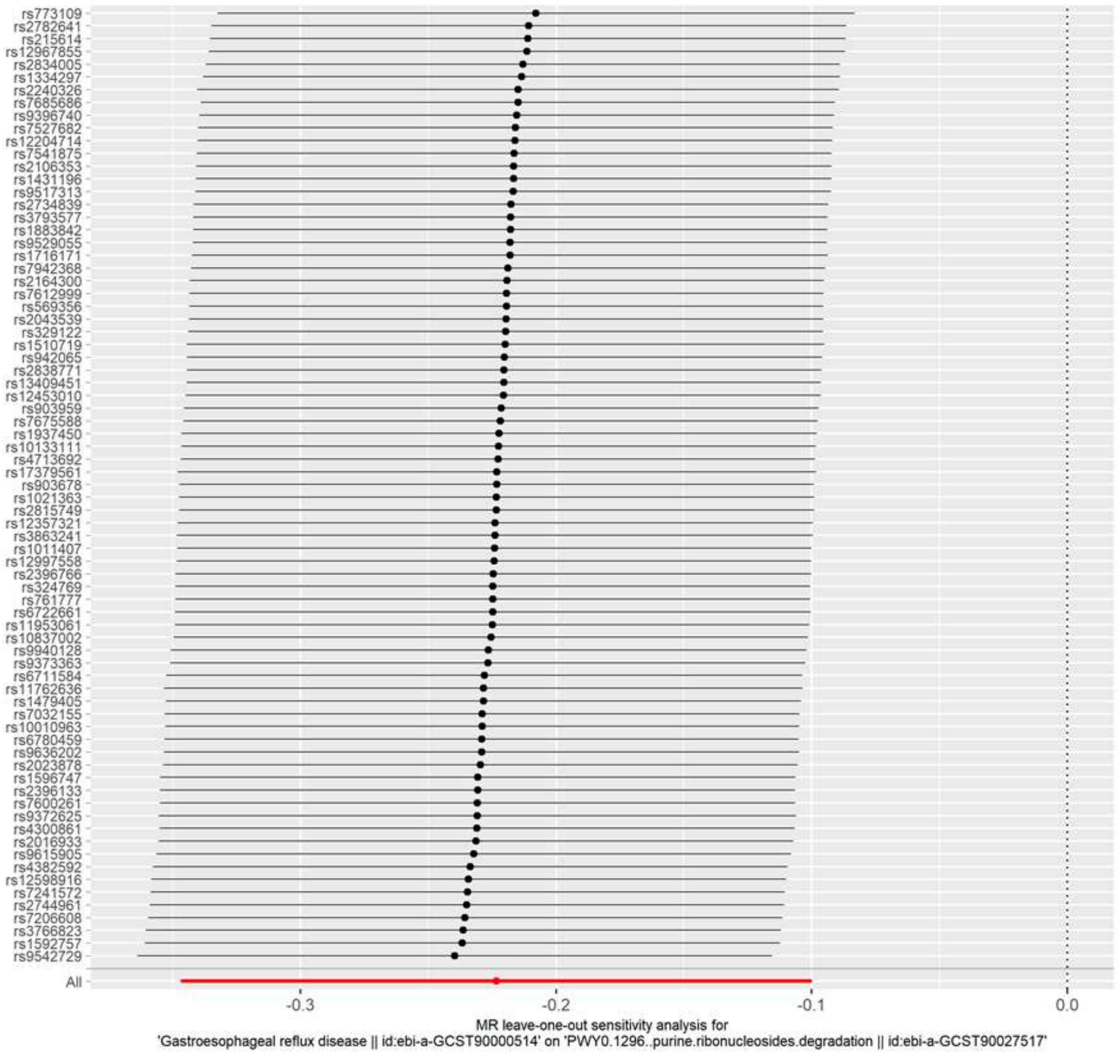

Supplementary Figure 6: Leave-one-out plot of SNPs related to GERD and gut bacterial pathway abundance (PWY0.1296..purine.ribonucleosides.degradation).

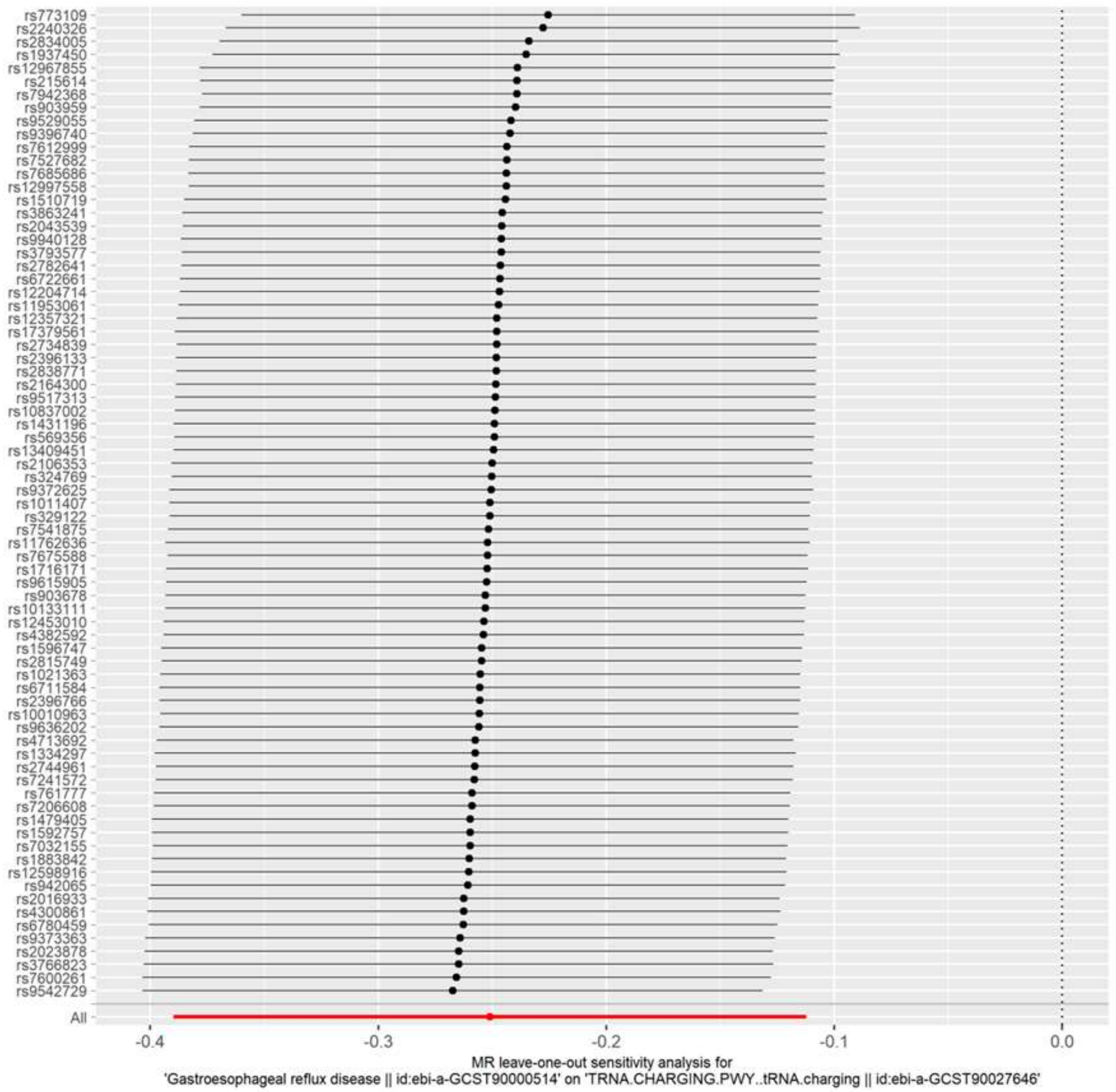

Supplementary Figure 7: Leave-one-out plot of SNPs related to GERD and gut bacterial pathway abundance (TRNA.CHARGING.PWY..tRNA.charging).

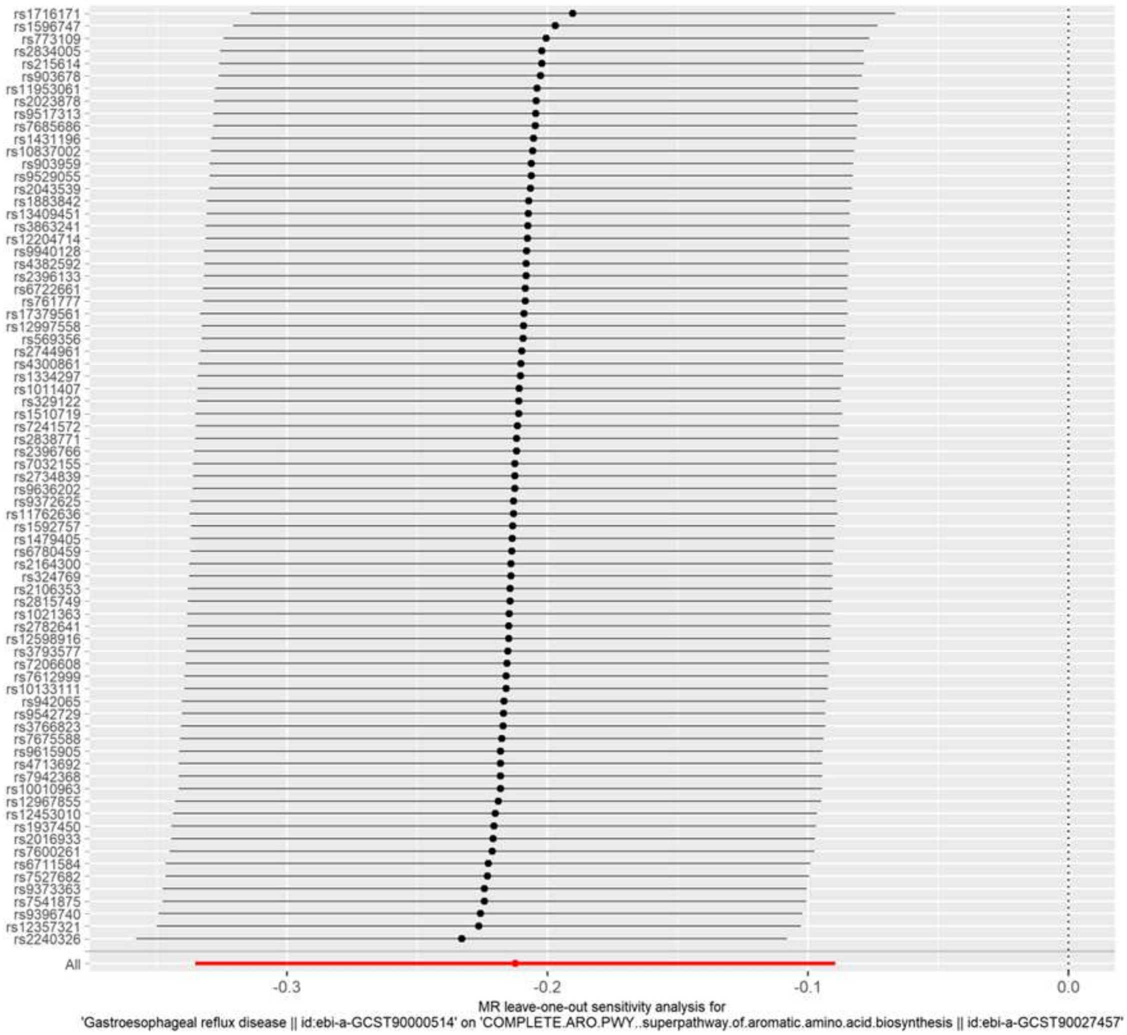

Supplementary Figure 8: Leave-one-out plot of SNPs related to GERD and gut bacterial pathway abundance (COMPLETE.ARO.PWY..superpathway.of.aromatic.amino.acid.biosynthesis).

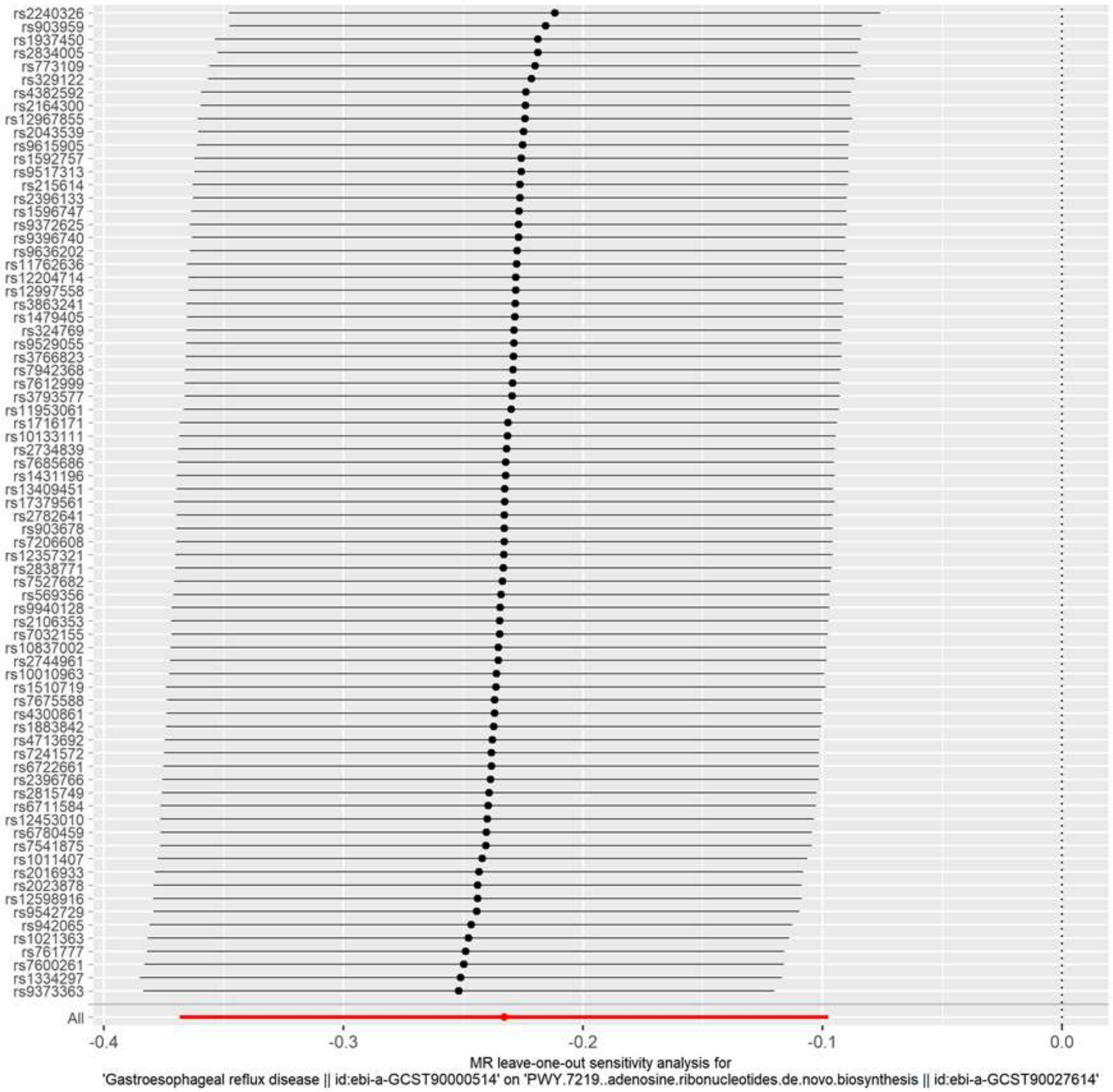

Supplementary Figure 9: Leave-one-out plot of SNPs related to GERD and gut bacterial pathway abundance (PWY.7219..adenosine.ribonucleotides.de.novo.biosynthesis).

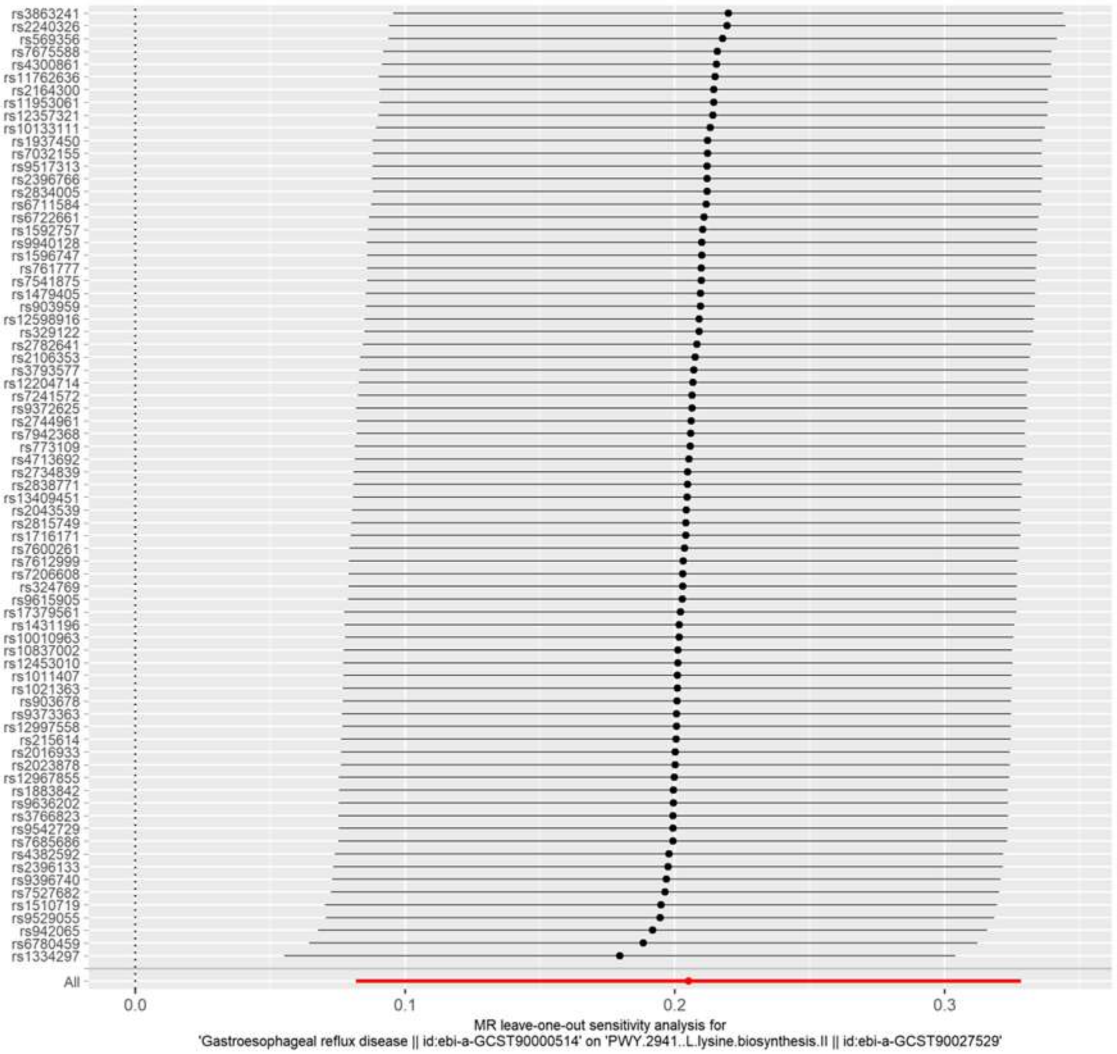

Supplementary Figure 10: Leave-one-out plot of SNPs related to GERD and gut bacterial pathway abundance (PWY.2941..L.lysine.biosynthesis.II).

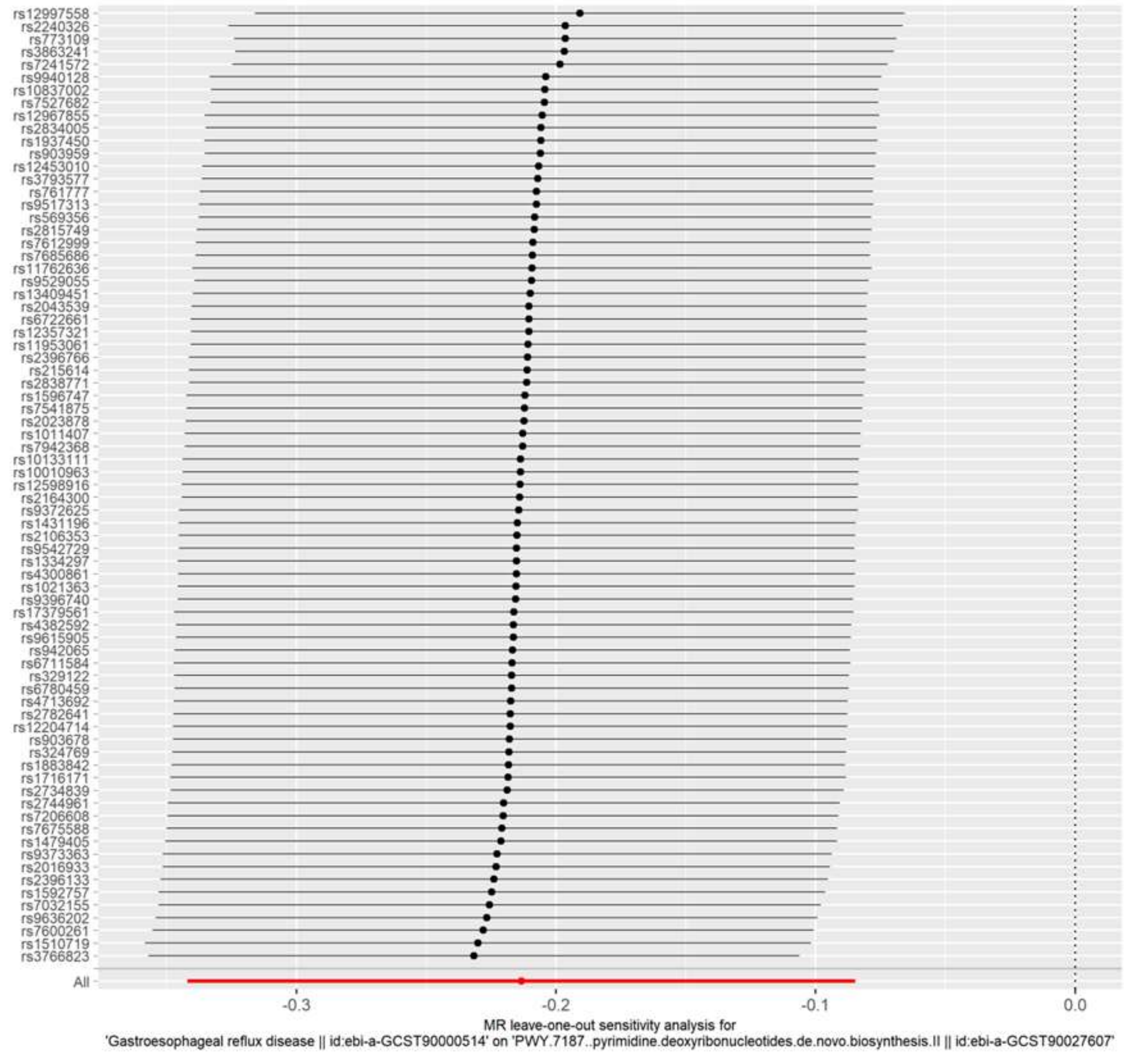

Supplementary Figure 11: Leave-one-out plot of SNPs related to GERD and gut bacterial pathway abundance (PWY.7187..pyrimidine.deoxyribonucleotides.de.novo.biosynthesis.II).

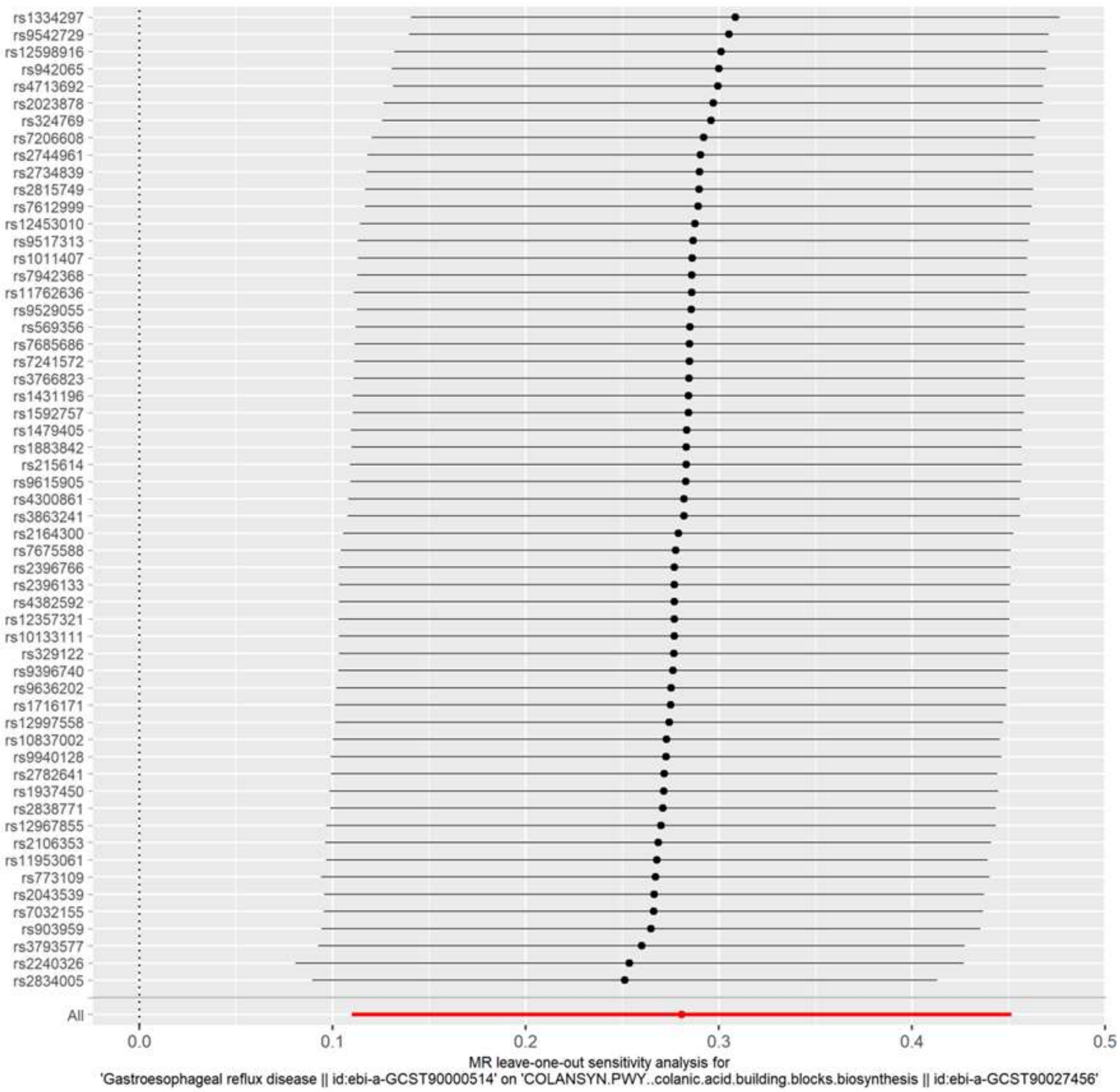

Supplementary Figure 12: Leave-one-out plot of SNPs related to GERD and gut bacterial pathway abundance (COLANSYN.PWY..colanic.acid.building.blocks.biosynthesis).

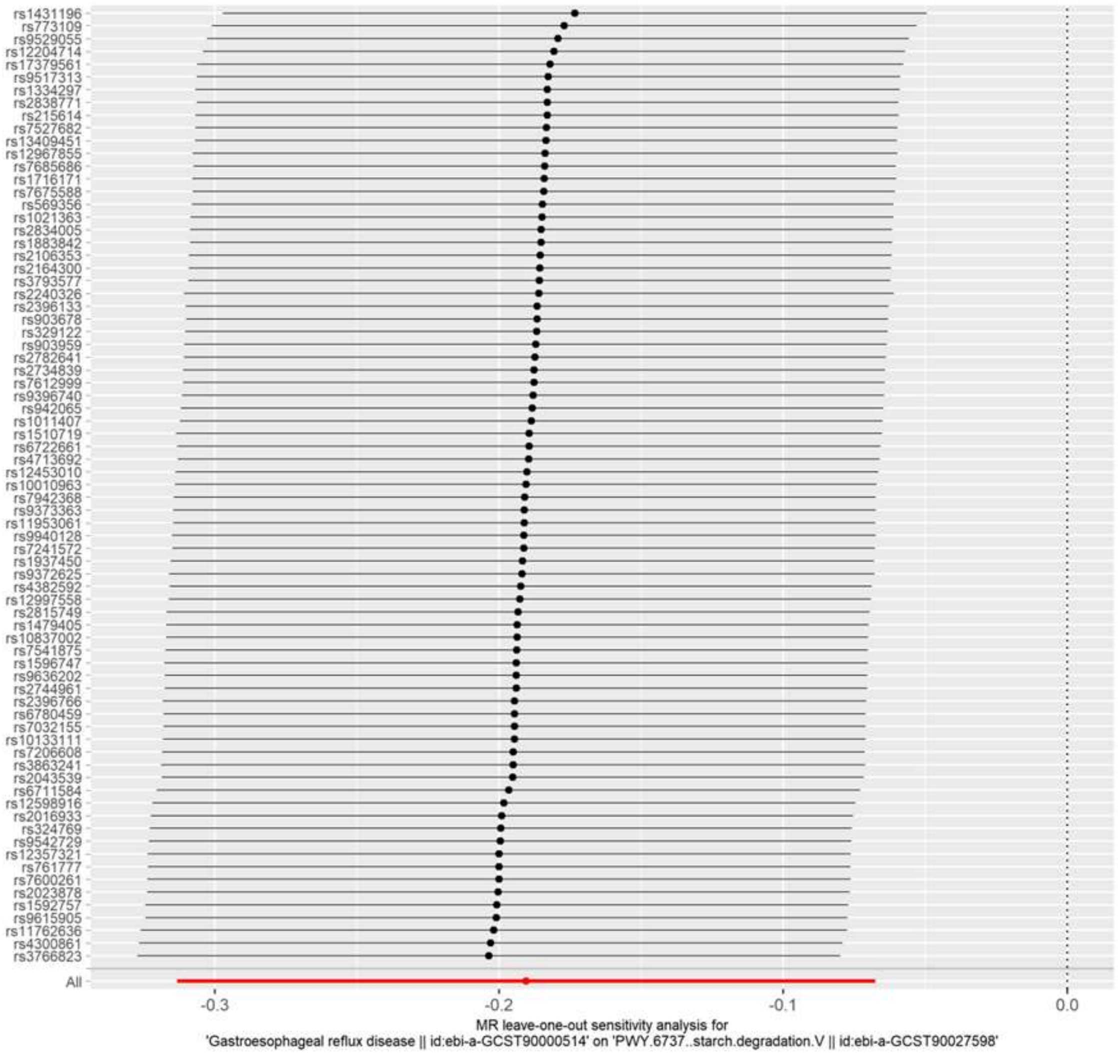

Supplementary Figure 13: Leave-one-out plot of SNPs related to GERD and gut bacterial pathway abundance (PWY.6737..starch.degradation.V).

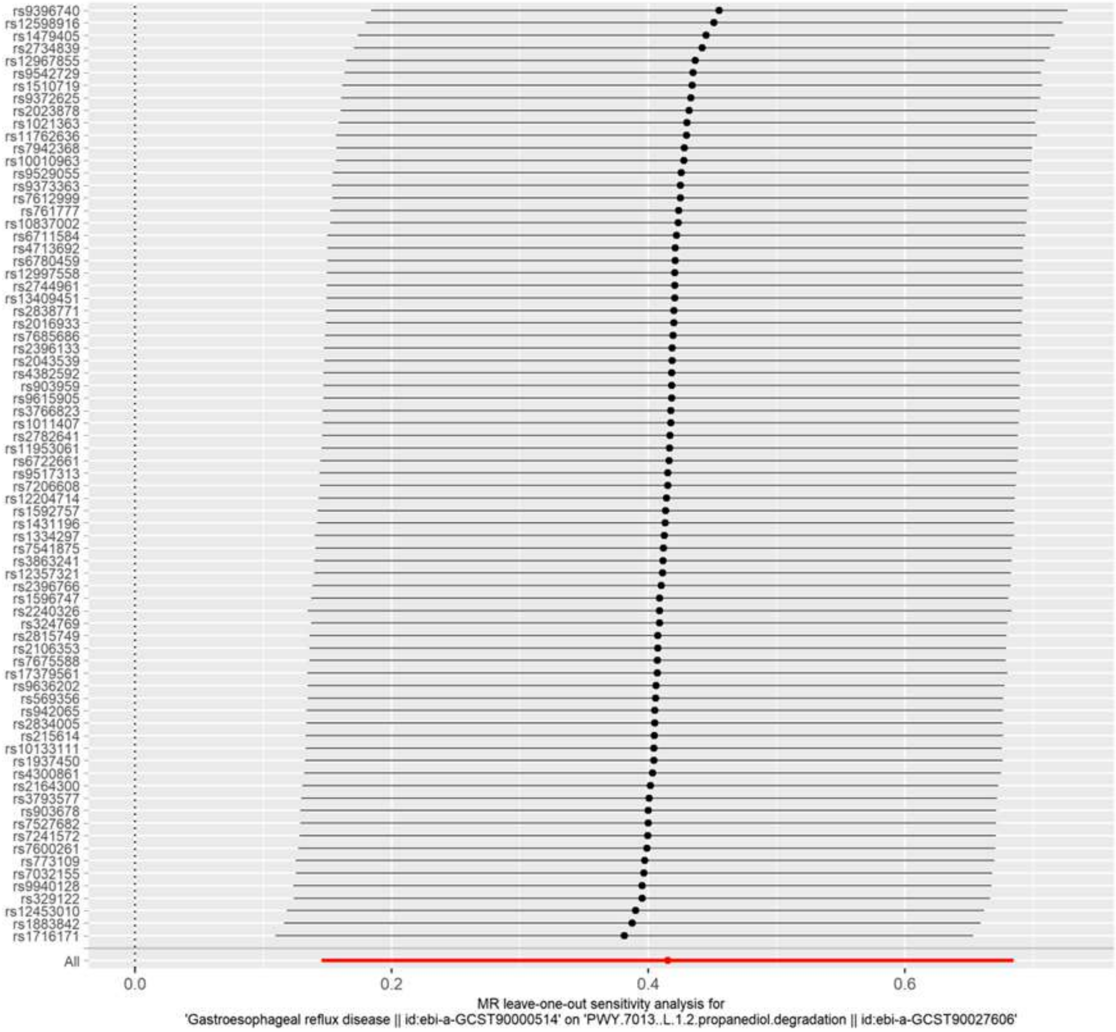

Supplementary Figure 14: Leave-one-out plot of SNPs related to GERD and gut bacterial pathway abundance (PWY.7013..L.1.2.propanediol.degradation).

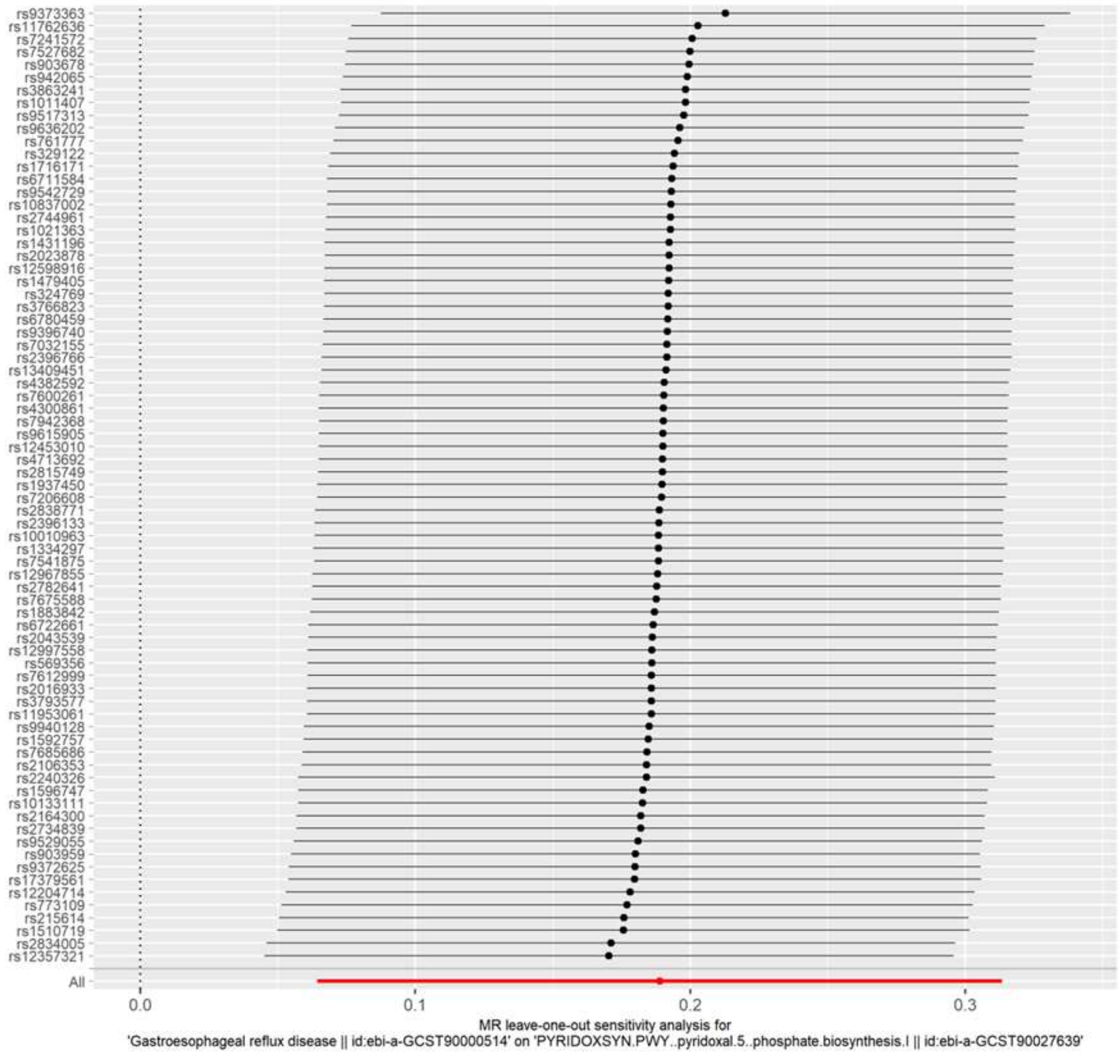

Supplementary Figure 15: Leave-one-out plot of SNPs related to GERD and gut bacterial pathway abundance (PYRIDOXSYN.PWY..pyridoxal.5..phosphate.biosynthesis.I).

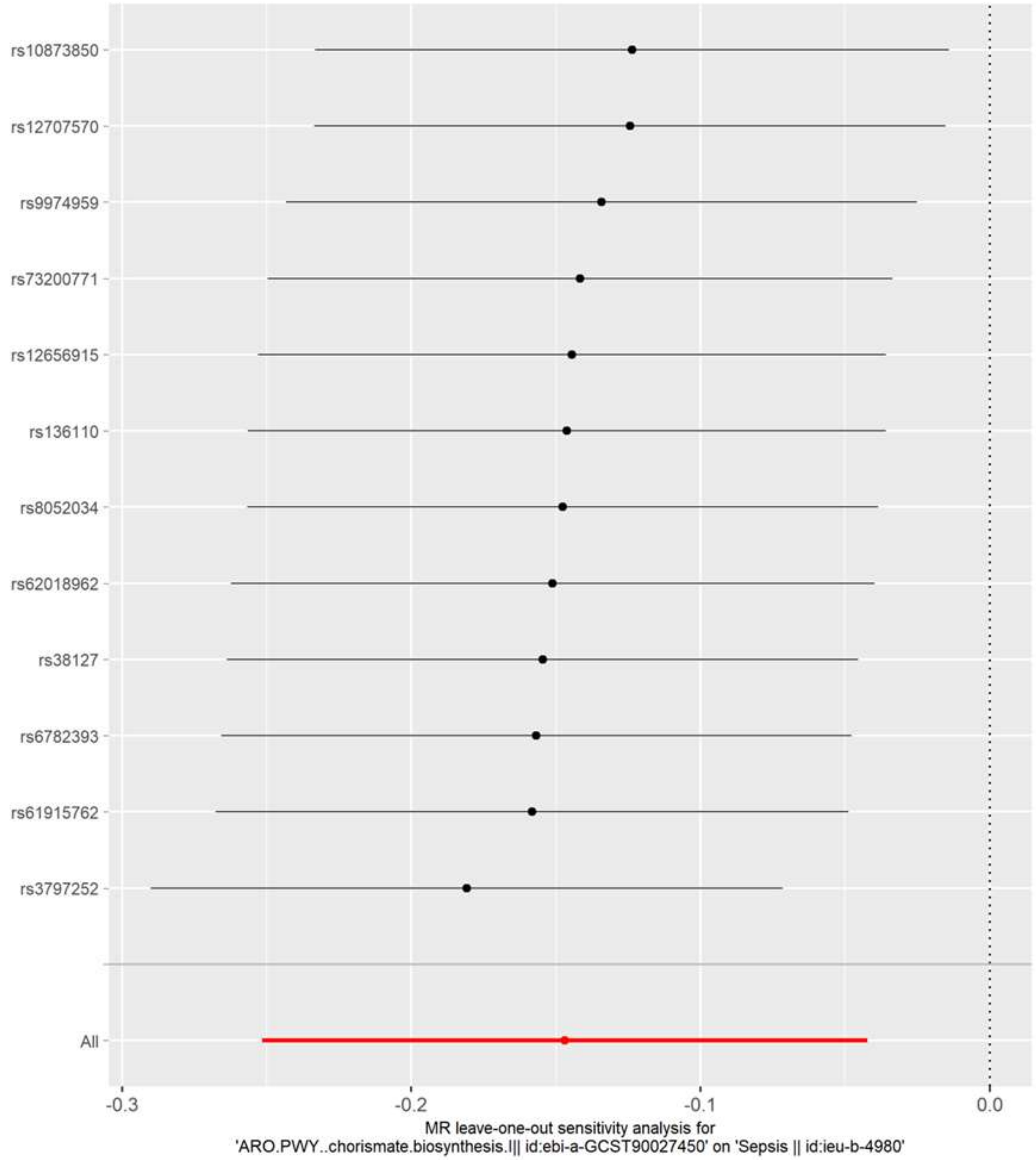

Supplementary Figure 16: Leave-one-out plot of SNPs related to gut bacterial pathway abundance (ARO.PWY..chorismate.biosynthesis.I) and sepsis.

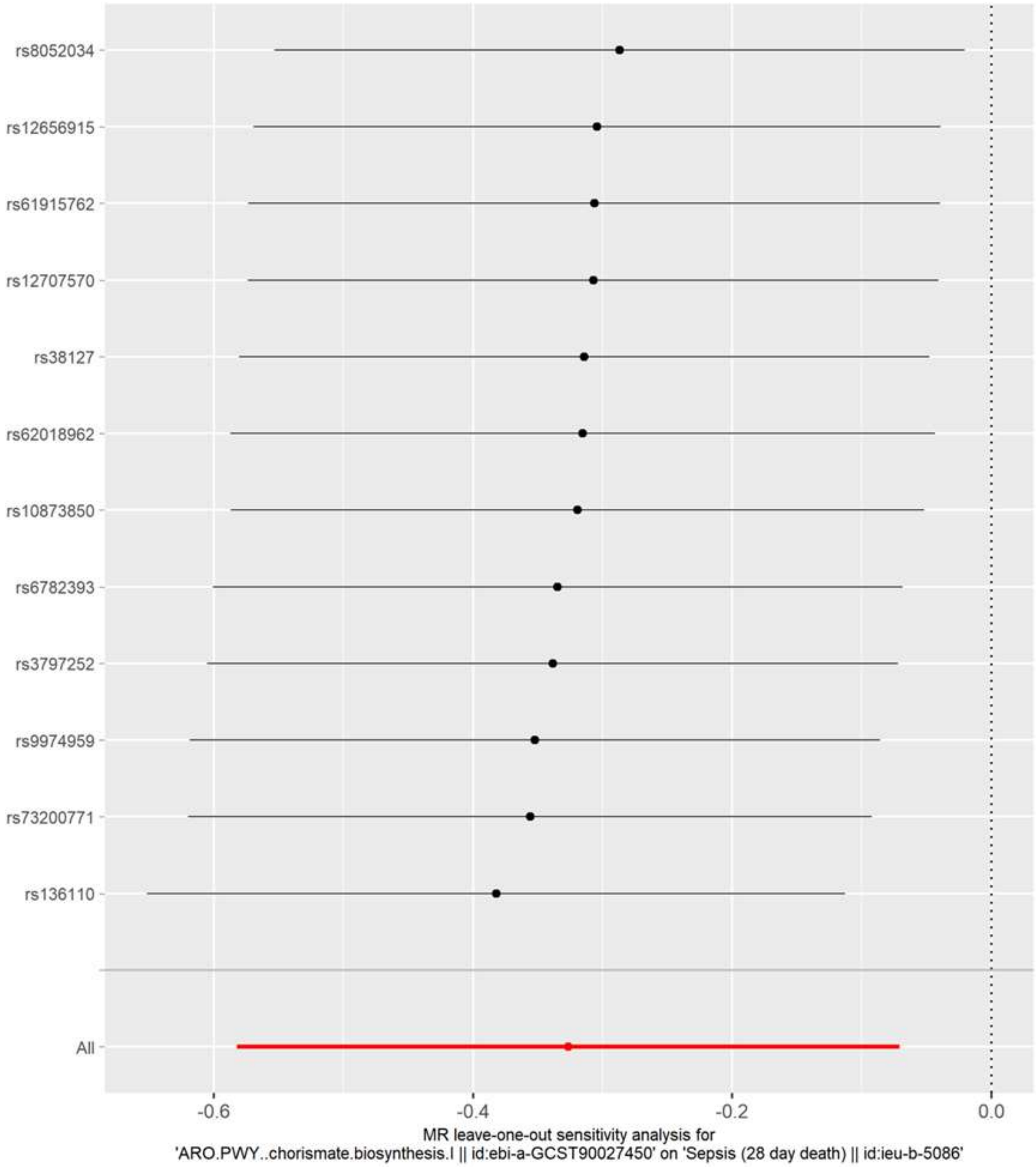

Supplementary Figure 17: Leave-one-out plot of SNPs related to gut bacterial pathway abundance (ARO.PWY..chorismate.biosynthesis.I) and sepsis(28 day death).

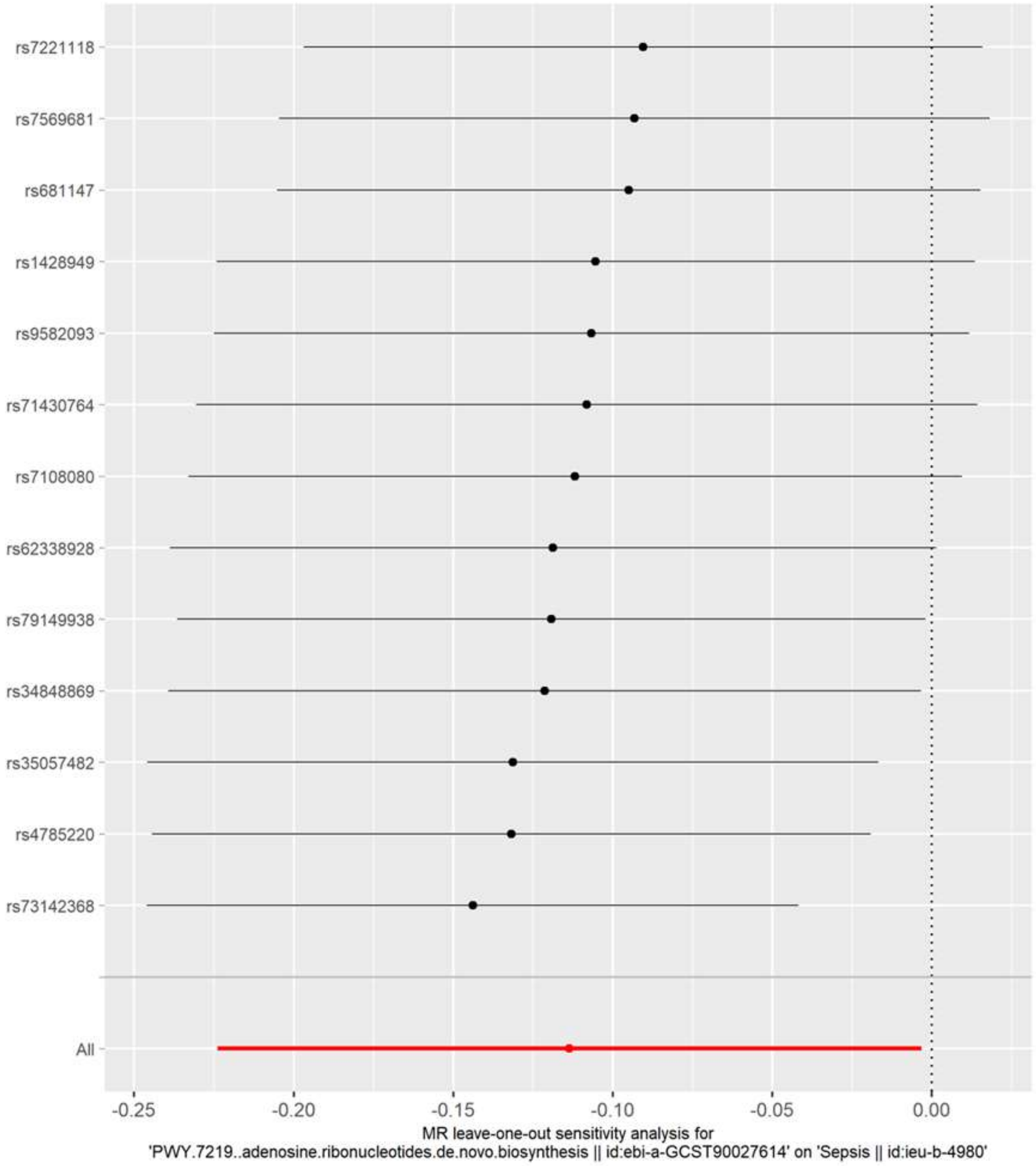

Supplementary Figure 18: Leave-one-out plot of SNPs related to gut bacterial pathway abundance (PWY.7219..adenosine.ribonucleotides.de.novo.biosynthesis) and sepsis.

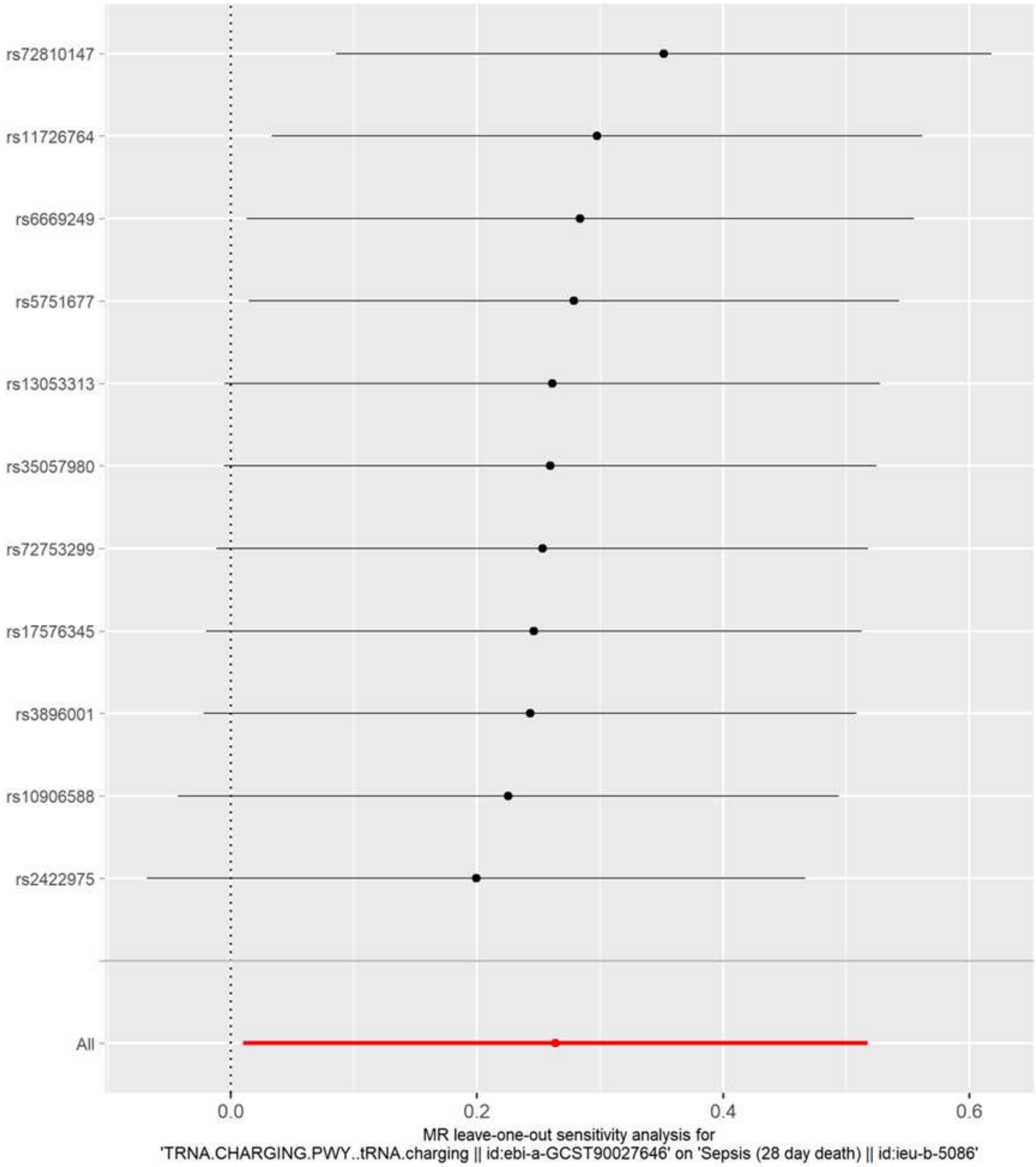

Supplementary Figure 19: Leave-one-out plot of SNPs related to gut bacterial pathway abundance (TRNA.CHARGING.PWY..tRNA.charging) and sepsis(28 day death).
