# Supplementary material for: Causal association between gastroesophageal reflux disease and sepsis, and the mediating role of gut bacterial abundance, a Mendelian randomization study
Source: Medicine (Baltimore). 2025 Feb 21;104(8):e41631. doi: 10.1097/MD.0000000000041631 (PMC11857025; doi:10.1097/MD.0000000000041631)
Supplement: Supplementary file 3 [file medi-104-e41631-s003.docx]

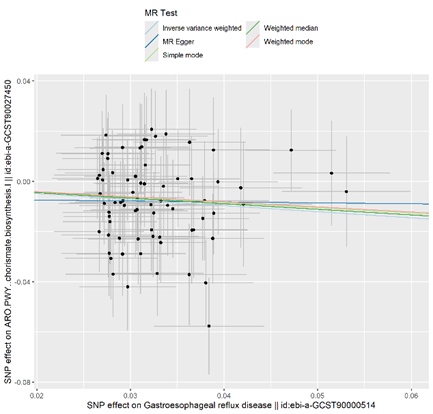


Supplementary File 1: Scatter plot showing the influence of GERD on gut bacterial pathway abundance (ARO.PWY..chorismate.biosynthesis.I).


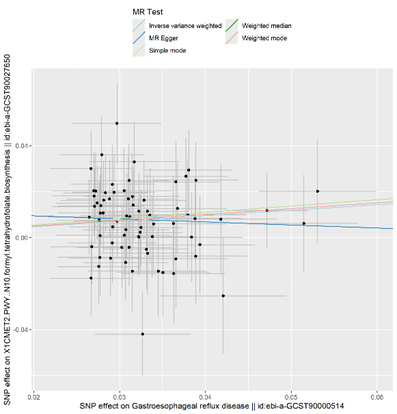


Supplementary File 2: Scatter plot showing the influence of GERD on gut bacterial pathway abundance (X1CMET2.PWY..N10.formyl.tetrahydrofolate.biosynthesis).


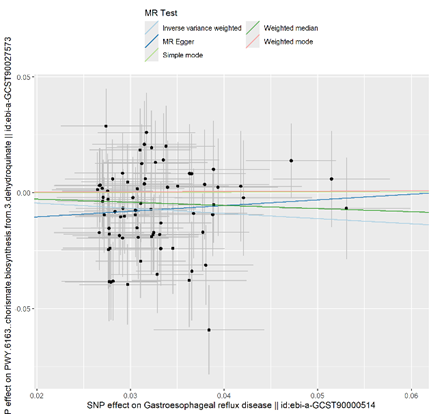


Supplementary File 3: Scatter plot showing the influence of GERD on gut bacterial pathway abundance (PWY.6163..chorismate.biosynthesis.from.3.dehydroquinate).


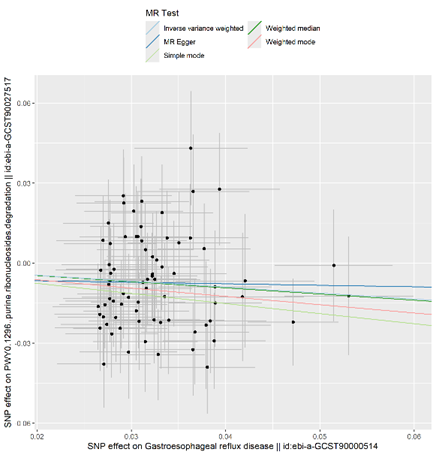


Supplementary File 4: Scatter plot showing the influence of GERD on gut bacterial pathway abundance (PWY0.1296..purine.ribonucleosides.degradation).


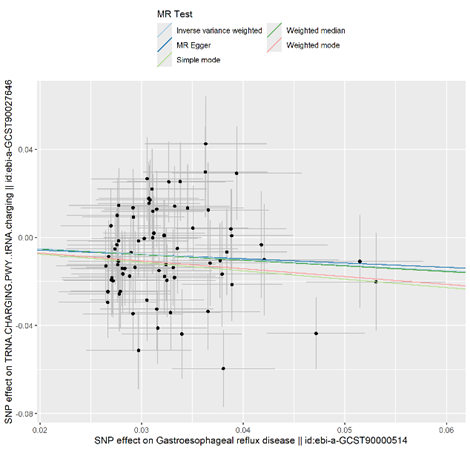


Supplementary File 5: Scatter plot showing the influence of GERD on gut bacterial pathway abundance (TRNA.CHARGING.PWY..tRNA.charging).


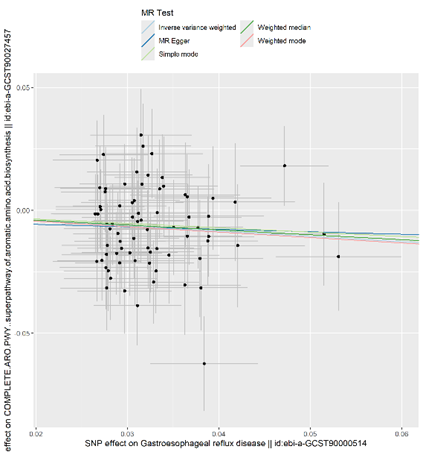


Supplementary File 6: Scatter plot showing the influence of GERD on gut bacterial pathway abundance (COMPLETE.ARO.PWY..superpathway.of.aromatic.amino.acid.biosynthesis).


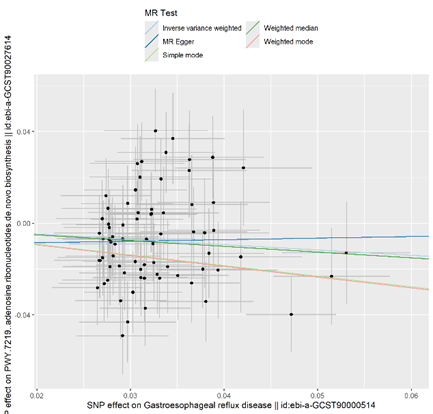


Supplementary File 7: Scatter plot showing the influence of GERD on gut bacterial pathway abundance (PWY.7219..adenosine.ribonucleotides.de.novo.biosynthesis).


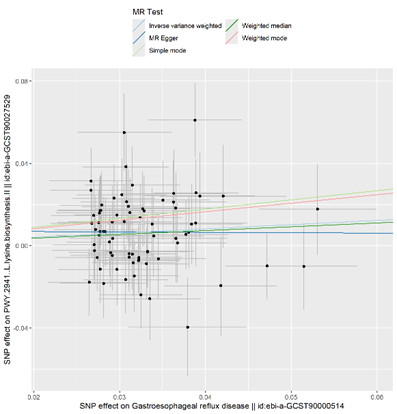


Supplementary File 8: Scatter plot showing the influence of GERD on gut bacterial pathway abundance (PWY.2941..L.lysine.biosynthesis.II).


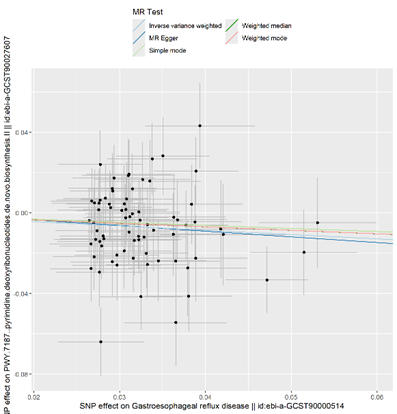


Supplementary File 9: Scatter plot showing the influence of GERD on gut bacterial pathway abundance (PWY.7187..pyrimidine.deoxyribonucleotides.de.novo.biosynthesis.II).


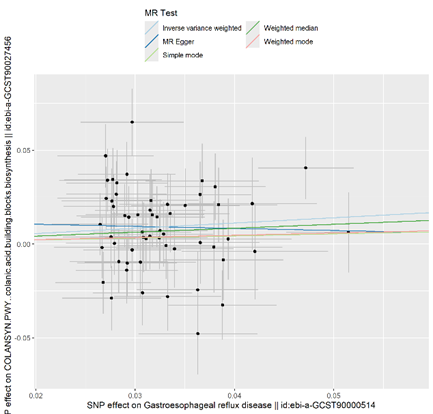


Supplementary File 10: Scatter plot showing the influence of GERD on gut bacterial pathway abundance (COLANSYN.PWY..colanic.acid.building.blocks.biosynthesis).


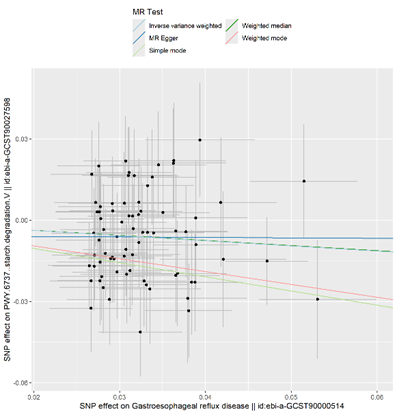


Supplementary File 11: Scatter plot showing the influence of GERD on gut bacterial pathway abundance (PWY.6737..starch.degradation.V).


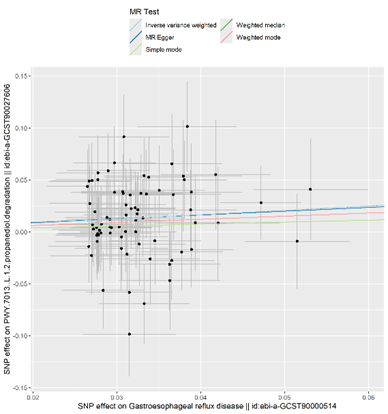


Supplementary File 12: Scatter plot showing the influence of GERD on gut bacterial pathway abundance (PWY.7013..L.1.2.propanediol.degradation).


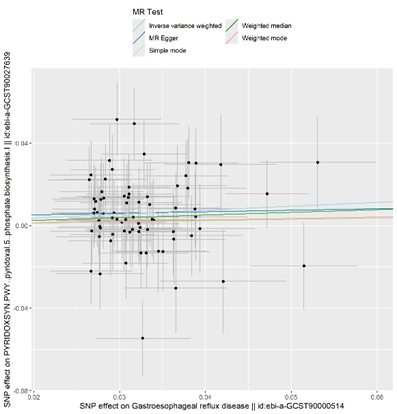


Supplementary File 13: Scatter plot showing the influence of GERD on gut bacterial pathway abundance (PYRIDOXSYN.PWY..pyridoxal.5..phosphate.biosynthesis.I).


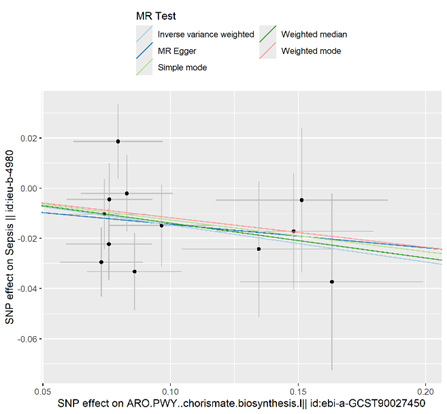


Supplementary File 14: Scatter plot showing the influence of gut bacterial pathway abundance (ARO.PWY..chorismate.biosynthesis.I) on sepsis.


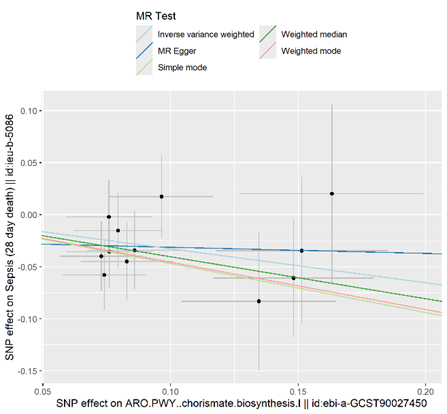


Supplementary File 15: Scatter plot showing the influence of gut bacterial pathway abundance (ARO.PWY..chorismate.biosynthesis.I) on sepsis(28 day death).


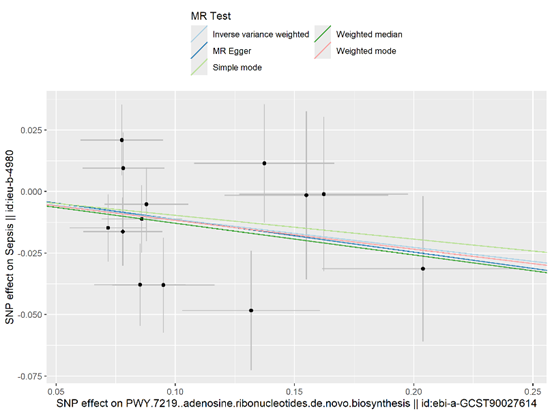


Supplementary File 16: Scatter plot showing the influence of gut bacterial pathway abundance (PWY.7219..adenosine.ribonucleotides.de.novo.biosynthesis) on sepsis.


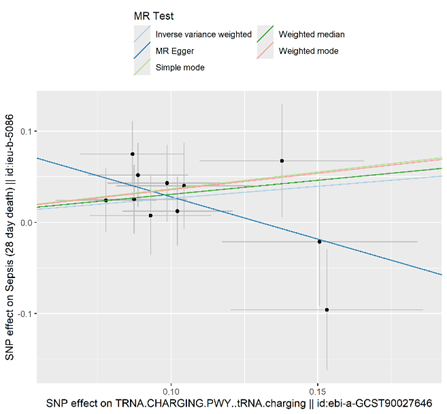


Supplementary File 17: Scatter plot showing the influence of gut bacterial pathway abundance (TRNA.CHARGING.PWY..tRNA.charging) on sepsis(28 day death).
